# Design of indole- and MCR-based macrocycles as p53-MDM2 antagonists

**DOI:** 10.3762/bjoc.15.45

**Published:** 2019-02-20

**Authors:** Constantinos G Neochoritis, Maryam Kazemi Miraki, Eman M M Abdelraheem, Ewa Surmiak, Tryfon Zarganes-Tzitzikas, Beata Łabuzek, Tad A Holak, Alexander Dömling

**Affiliations:** 1 Department of Drug Design, University of Groningen, Antonius Deusinglaan 1, 9700 AD Groningen, The Netherlands; 2 Chemistry Department, Tarbiat Modares University, P.O. Box 14155-4838, Tehran, Iran; 3 Faculty of Chemistry, Jagiellonian University, Gronostajowa 2, 30-387 Krakow, Poland

**Keywords:** ^1^H,^15^N HSQC NMR, indole, macrocycles, multicomponent, p53-MDM2, Ugi reaction

## Abstract

Macrocycles were designed to antagonize the protein–protein interaction p53-MDM2 based on the three-finger pharmacophore F^19^W^23^L^25^. The synthesis was accomplished by a rapid, one-pot synthesis of indole-based macrocycles based on Ugi macrocyclization. The reaction of 12 different α,ω-amino acids and different indole-3-carboxaldehyde derivatives afforded a unique library of macrocycles otherwise difficult to access. Screening of the library for p53-MDM2 inhibition by fluorescence polarization and ^1^H,^15^N HSQC NMR measurements confirm MDM2 binding.

## Introduction

Macrocycles are the chemical entities that are consisting of a 12-membered or even bigger ring. It is estimated that 3% of the known natural products consists of a macrocyclic ring [1–5]. Compared to macrocycles in synthetic molecules, the aforementioned occurrence is still over proportional; for that reason, these compounds have delighted scientists worldwide due to their special physicochemical properties, their roles in biological systems and the associated synthetic challenges [6–7]. However, only few synthetic methods allow for the convergent and fast access to a large macrocyclic chemical space [8–10]; most of the times their synthesis is complex, multistep and sequential [11–12]. For this reason a great effort is ongoing to utilize multicomponent reactions for the synthesis of macrocycles [8,13–25].

**Scheme 1:**
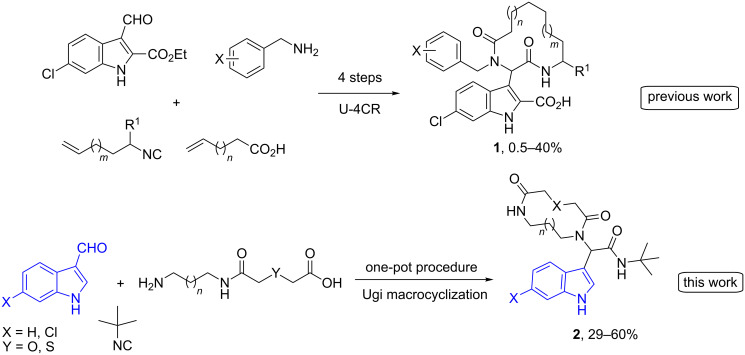
MCR approach to indole-based macrocycles; a more effective strategy is proposed in this work, based on α,ω-amino acids and an Ugi macrocyclization.

The p53 protein is a well-studied protein which has a leading role in protecting our organism from cancer. It was found that most of the human cancers have either mutated the p53 itself or the p53 pathway is inhibited. The latter group of tumors retains the wild type p53 (wt-p53) but its pathway is inactivated by negative regulators, mainly the MDM2 and MDMX proteins. Thus, the design and synthesis of an inhibitor of the MDM2–p53 interaction could enable p53 and reverse tumor formation [26–28]. Based on our knowledge to antagonize the oncogenic protein–protein interaction p53–MDM2 [23,29–40] we designed macrocyclic inhibitors in continuation of our previous work [13,23]. Herein, an indole-based macrocycle synthesis is reported in a one-pot fashion based on Ugi macrocyclization with readily available α,ω-amino acids. Moreover, in continuation of our efforts in the design and synthesis of macrocycles targeting the p53–MDM2 interaction demonstrating the potential of these indole-based macrocycles, a subset of them was screened searching for MDM2 inhibitors. Compared to our previous indole-based macrocycles **1** following a different strategy (employing a classical Ugi-4C as the key reaction) [23], this one-pot Ugi macrocyclization leading to macrocycles **2** offers speed (one-pot procedure with one purification step), much better yields, no need of expensive catalysts as in ring-closing metathesis (RCM) reaction and higher complexity/diversity on the macrocyclic ring, e.g., insertion of heteroatoms that could improve the ADMET properties (Scheme 1) [4].

## Results and Discussion

### Synthesis

**Scheme 2:**
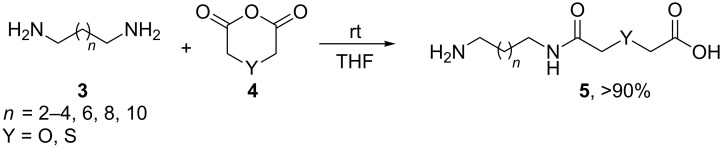
Reaction of unprotected diamines **3** with cyclic anhydrides **4** at rt affording α,ω-amino acids **5** in quantitative yields.

Based on our previous studies [13], unprotected diamines **3** were reacted in one-step with cyclic anhydrides **4** at rt affording the appropriate α,ω-amino acids **5** in excellent yields (see Supporting Information File 1). Elongated diamines (*n* = 2–4, 6, 8 and 10) and cyclic anhydrides that bear a heteroatom in the 4-position as oxygen or sulfur (Y = O, S, Scheme 2) were employed in order to enhance the diversity of our macrocycles [4]. Thus, in a parallel way, we readily synthesized 12 different amino acids which were subsequently subjected to the Ugi macrocyclization.

**Scheme 3:**
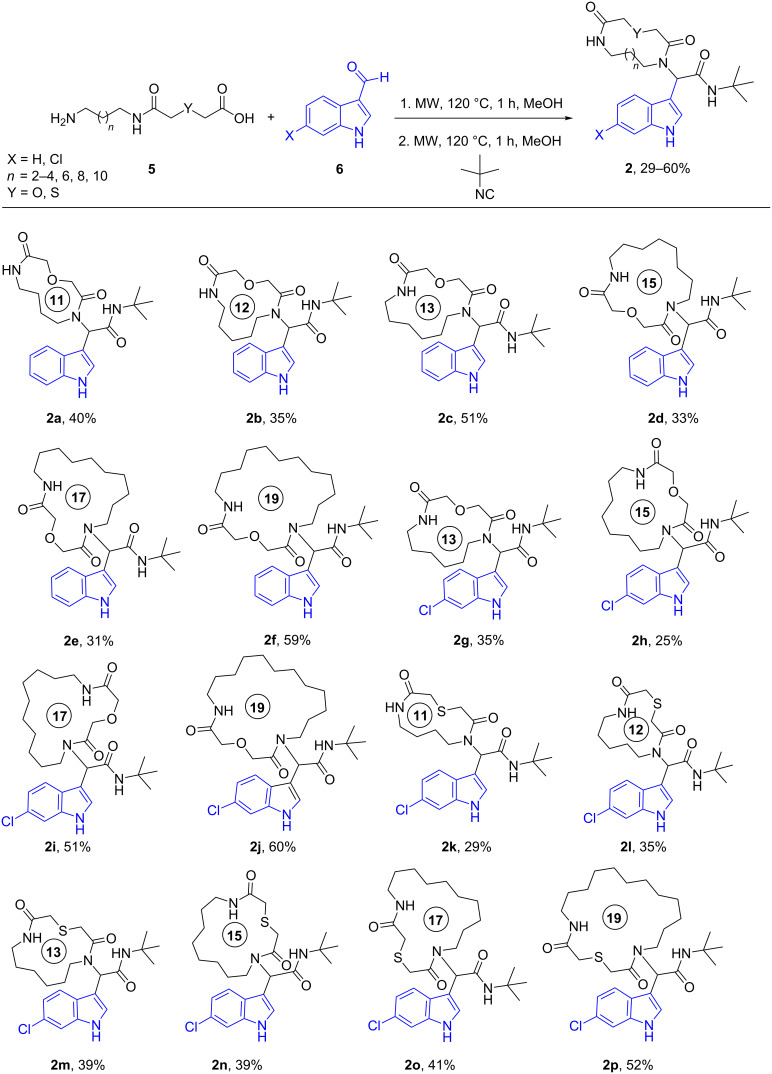
Ugi macrocyclization in a one-pot fashion and synthesis of diverse indole-based macrocycles. The circle depicts the size number of the macrocycle.

After quite some optimization, we improved the Ugi-macrocyclization procedure compared to our previous findings utilizing microwave irradiation (see Supporting Information File 1); Firstly, the corresponding amino acid was irradiated with indole-3-carboxaldehyde derivatives **6** using MeOH as solvent (5 mL) at 120 °C for 1 h. Then, *tert*-butyl isocyanide was added, diluted with more MeOH and irradiated again the reaction mixture at 120 °C for an additional 1 h in a final concentration of 0.1 M (Scheme 3). By this way, a rapid, one-pot access to macrocycles **2a–p** was achieved otherwise very difficult to synthesize in relatively good yields (29–60%). 16 different indole-based macrocycles were synthesized with their size varying from 11–13, 15, 17 and 19 atoms (Scheme 3).

### Biological evaluation

**Figure 1:**
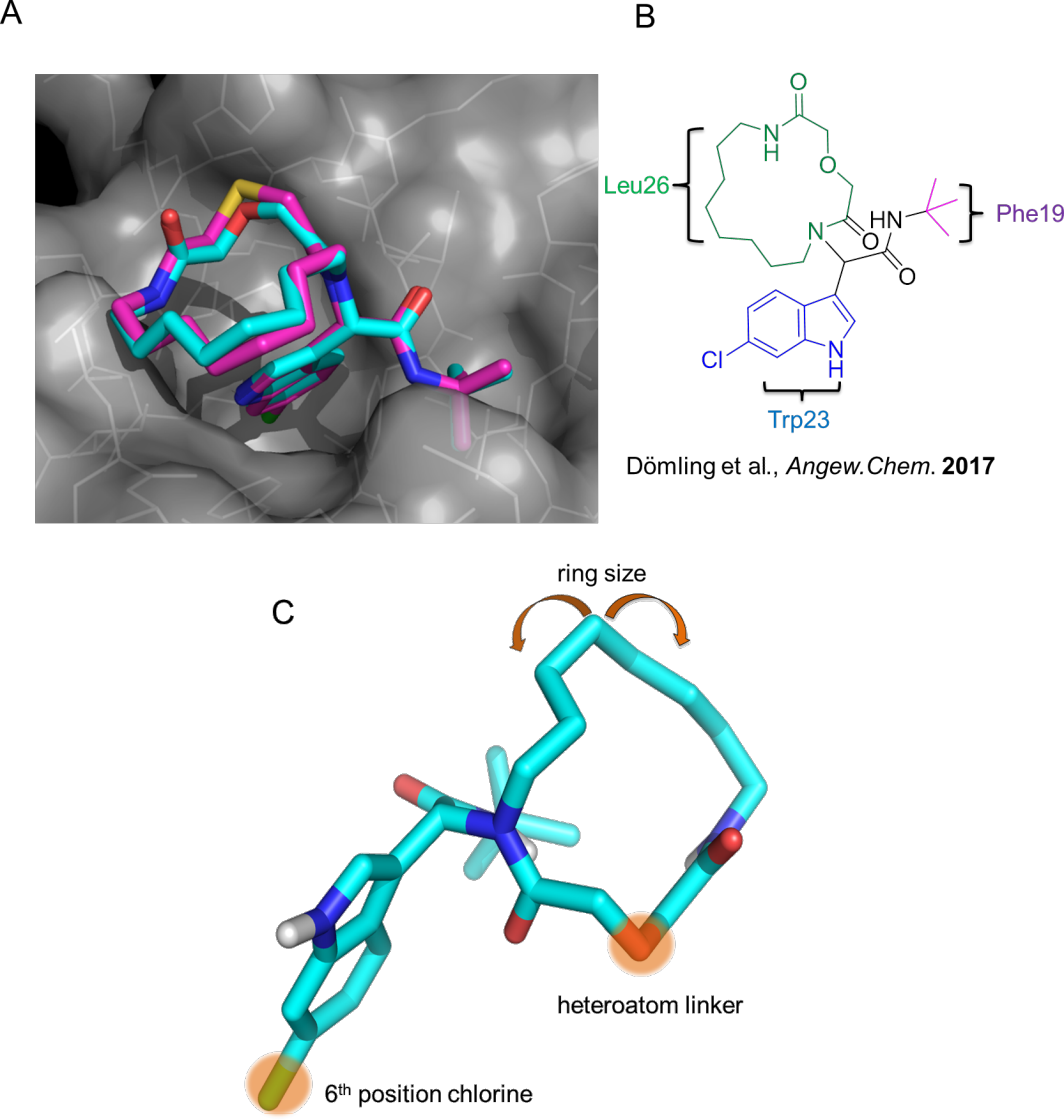
(A) Modeling of the macrocycle **2h** (cyan sticks) and **2n** (magenta sticks) into the MDM2 receptor (PDB ID: 1YCR); (B) 2D structure of **2h** with the substituents targeting the subpockets of MDM2; (C) Analysis of the synthesized macrocycles probing the subpockets of MDM2 and expansion of the chemistry compared to previous studies [13].

Our previously introduced three-point pharmacophore model on mimicking the hot triad (Phe19, Trp23 and Leu26, F^19^W^23^L^26^) was the basis of the evaluation of the current derivatives as potent inhibitors [33]. The indole moiety could be used not only to constrain the two other substituents but also as an “anchor” mimicking the Trp23. The bulky *tert*-butyl group would mimic the Phe19 and the macrocyclic ring would fill the Leu26 sub-pocket as shown by our docking studies (Figure 1A,B, Figure S4 in Supporting Information File 1). Thus, extending our previous work [13], the Leu26 subpocket was probed by utilizing the different ring sizes and the different heteroatoms (oxygen or sulfur) of our macrocyclic library. In addition, the influence of the chlorine atom in the 6-position of the indole ring (Figure 1C) was examined. Macrocycles **2a–j** consist of an oxygen linker whereas **2g–j** bear also a chlorine atom in the 6-position in the indole ring. Macrocycles **2k–p** incorporate both a sulfur linker and the chlorine on the indole ring (Scheme 3).

**Figure 2:**
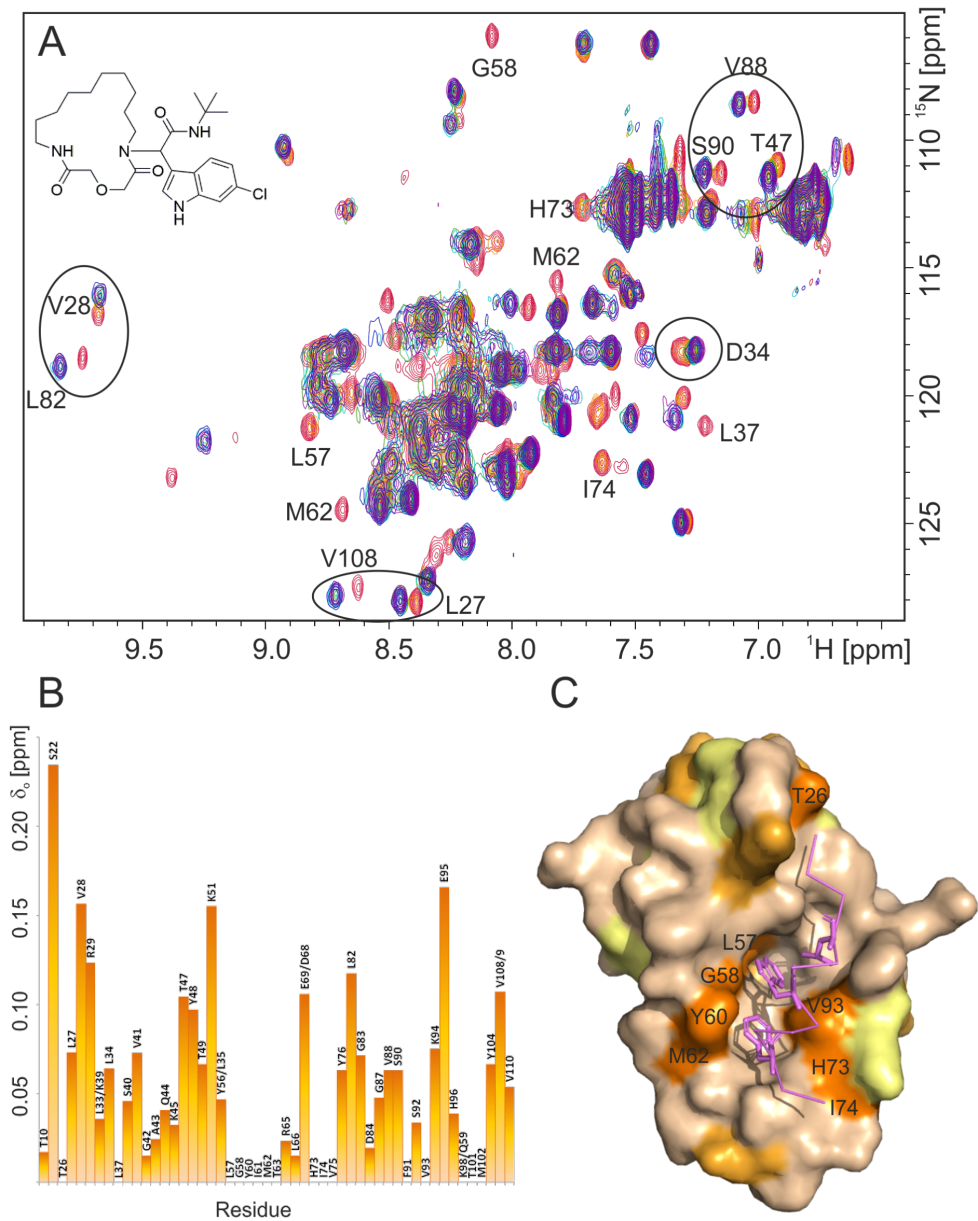
(A) Overlay of ^1^ H,^15^N-HSQC spectra of the reference MDM2 (red) and the titration steps with the **2i** inhibitor. MDM2/**2i** ratios 4:1 (orange), 4:2 (yellow), 4:3 (green), 1:1 (light blue), 1:2 (blue), 1:5 (purple). Examples of most perturbed residues are labeled on the spectrum. (B) Normalized chemical shift perturbations (δ_o_) of MDM2 residue (calculated according to Stoll et al. [41]). Residue with δ_o_ equal 0 are either despairing from MDM2 spectrum upon titration or cannot be identified. (C) Chemical shift perturbations plotted onto the structure of MDM2 (wheat); orange (despairing – indicating stronger binding), light orange (>0.1 ppm), yellow (0.05–0.1 ppm). Residues which disappear upon titration experiment are labeled on the Mdm2 surface.

In order to exclude false positive hits, two biorthogonal assays were chosen; ^1^H,^15^N HSQC NMR and fluorescence polarization (FP, Table 1). FP assay was employed to determine the inhibitory affinities (*K*_i_) of the derivatives against MDM2 as previously described [36]. Besides **2h** (*K*_i_ = 2.3 μΜ, *K*_d_ = 12.1 μΜ), it was shown that **2i** demonstrated a promising activity with a *K*_i_ of 5.5 μΜ. Furthermore, ^1^H,^15^N HSQC showed a *K*_d_ of 4.8 μΜ (Table 1, Figure 2). Moreover, macrocycles **2g** and **2n** demonstrated a *K*_d_ of 9 μΜ and 17 μΜ, respectively (Table 1). With this preliminary analysis, it was found that a ring size of 15–17 atoms and an oxygen as the heteroatom linker improves the binding affinity. All the active macrocycles have a 6-chloro-substituted indole core. It is well established that at the bottom of the Try23 pocket a hydrophobic small subpocket exists which is formed by Phe86, Ile103, Leu82 and Leu57. This pocket when filled with a smaller hydrophobic substituent such as -Cl boosts the inhibitor activity in accordance with literature [33].

**Table 1:** Measurement of *K*_i_ and *K*_d_ of the selected macrocycles based on FP and ^1^H,^15^N HSQC NMR assays, respectively.^a^

Entry	Name	Structure	*K*_i_ MDM2 [µM]	*K*_d_ MDM2 [µM]

1	**2h**	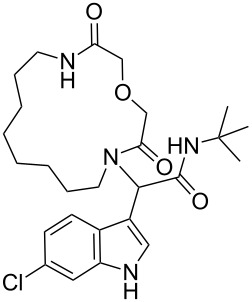	2.3	12.1 ± 8.5
2	**2i**	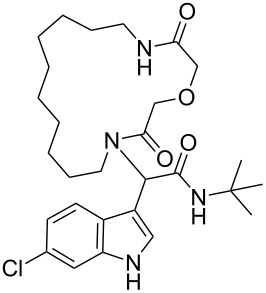	5.5	4.8 ± 1.5
3	**2n**	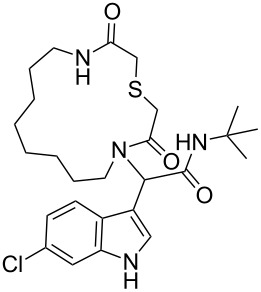	316	17.2 ± 3.8
4	**2g**	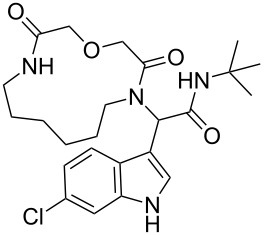	n.a.	8.9 ± 1.2

^a^n.a. no activity against MDM2 protein. *K*_i_ and *K*_d_ values were calculated based on fluorescence polarization binding and ^1^H,^15^N HSQC NMR assay, respectively.

## Conclusion

We effectively synthesized p53-MDM2 antagonists based on an artificial macrocyclic scaffold. 16 different derivatives were obtained and screened. The aforementioned artificial macrocycles combine the indole ring, a motif found in many bioactive molecules with the drug-like properties of a non-peptide macrocycle. We hypothesize that these chimeric derivatives of an indole and a macrocycle will offer new potential on specific PPIs and other postgenomic targets as it was demonstrated with the p53-MDM2 interaction.

## Supporting Information

File 1 Experimental procedures, analytical data, NMR spectra, fluorescence polarization binding assays, ^1^H,^15^N HSQC NMR spectra of ^15^N-labeled MDM2 and computational modeling studies.
