# Supplementary material for: Design of indole- and MCR-based macrocycles as p53-MDM2 antagonists
Source: Beilstein J Org Chem. 2019 Feb 20;15:513–20. doi: 10.3762/bjoc.15.45 (PMC6404402; doi:10.3762/bjoc.15.45)
Supplement: File 1 — Experimental procedures, analytical data, NMR spectra, fluorescence polarization binding assays, 1H,15N HSQC NMR spectra of 15N-labeled MDM2 and computational modeling studies. [file Beilstein_J_Org_Chem-15-513-s001.pdf]

BEILSTEIN JOURNAL OF ORGANIC CHEMISTRY

Supporting Information

for

Design of indole- and MCR-based macrocycles as p53-MDM2 antagonists

Constantinos G. Neochoritis, Maryam Kazemi Miraki, Eman M. M. Abdelraheem, Ewa Surmiak, Tryfon Zarganes-Tzitzikas, Beata Łabuzek, Tad A. Holak and Alexander Dömling

Beilstein J. Org. Chem. **2019,** *15,* 513–520. doi:10.3762/bjoc.15.45

Experimental procedures, analytical data, NMR spectra, fluorescence polarization binding assays, $^{1}H,^{15}N$ HSQC NMR spectra of ^{15}N-labeled MDM2 and computational modeling studies

License and Terms: This is a supporting information file under the terms of the Creative Commons Attribution License (http://creativecommons.org/licenses/by/4.0). Please note that the reuse, redistribution and reproduction in particular requires that the authors and source are credited.
The license is subject to the *Beilstein Journal of Organic Chemistry* terms and conditions: (https://www.beilstein-journals.org/bjoc)

Table of Contents

1. Experimental materials and methods S2

2. Synthetic procedures and analytical data S4

3. Exemplary copies of NMR and MS data of novel compounds S16

4. Fluorescence polarization binding assays S22

5. Titration of ^{15}N-labeled MDM2 with inhibitors S23

6. Computational modeling of macrocycles **2h**, **2i** and **2n** S27

References S28

1. Experimental materials and methods

1.1. Synthesis and analysis

All the reagents and solvents were purchased from Sigma-Aldrich, AK Scientific, Fluorochem, Abcr GmbH, Acros and were used without further purification. All microwave irradiation reactions were carried out in a Biotage Initiator™ Microwave Synthesizer. Thin-layer chromatography was performed on Millipore precoated silica gel plates (0.20 mm thick, particle size 25 µm). Nuclear magnetic resonance spectra were recorded on a Bruker Avance 500 spectrometers {^{1}H NMR (500 MHz), ^{13}C NMR (126 MHz)}. Chemical shifts for ^{1}H NMR were reported as δ values and coupling constants were in hertz (Hz). The following abbreviations were used for spin multiplicity: s = singlet, br s = broad singlet, d = doublet, t = triplet, q = quartet, quin = quintet, dd = double of doublets, ddd = double doublet of doublets, m = multiplet. Chemical shifts for ^{13}C NMR were reported in ppm relative to the solvent peak. Flash chromatography was performed on a Reveleris® X2 Flash Chromatography, using Grace® Reveleris Silica flash cartridges (12 gram). Mass spectra were measured on a Waters Investigator Supercritical Fluid Chromatograph with a 3100 MS Detector (ESI) using a solvent system of methanol and CO_2 on a Viridis silica gel column (4.6 × 250 mm, 5 µm particle size) or Viridis 2-ethylpyridine column (4.6 × 250 mm, 5 µm particle size). High resolution mass spectra were recorded using a LTQ-Orbitrap-XL (Thermo) at a resolution of 60000@m/z400.

1.2. Protein expression and purification

Fragment of the N-terminal domain of human MDM2 (residues 1-118) was cloned into the pET-20 (Novagen) and expressed in *E. coli* strain BL21-CodonPlus(DE3)-RIL as described previously.[1] In brief, cells were cultured at 37 °C. Protein expression was induced with 1 mM IPTG at OD600 of 0.8 and cultured for additional 5 h at 37 °C. Cells were collected by centrifugation and lysed by sonication. Inclusion bodies were collected by centrifugation, washed with PBS containing 0.05% Triton-X100 and subsequently solubilized in 6 M guanidine hydrochloride in 100 mM Tris-HCl, pH 8.0, containing 1 mM EDTA and 10 mM β-mercaptoethanol. The protein was dialyzed against 4 M guanidine hydrochloride pH 3.5 supplemented with 10 mM β-mercaptoethanol. Following, the protein was refolded by dropwise addition into 10 mM Tris-HCl, pH 7.0, containing 1 mM EDTA and 10 mM β-mercaptoethanol and slow mixing overnight at 4 °C. Ammonium sulfate was added to the final concentration of 1.5 M and the refolded protein was recovered on Butyl Sepharose 4 Fast Flow (GE Healthcare). The protein was eluted using 100 mM Tris-HCl pH 7.2 containing 5 mM β-mercaptoethanol and further purified by gel filtration on HiLoad 16/600 Superdex75 (GE

Healthcare) in 50 mM phosphate buffer pH 7.4 containing 150 mM NaCl and 5 mM DTT (FP/NMR buffer).

1.3. Fluorescence polarization assay

All FP measurements were performed using Tecan Infinite® 200 PRO plate reader. The assay was conducted in 50 mM NaCl, 10 mM Tris pH 8.0, 1 mM EDTA containing 5% DMSO. To determine the optimal concentration of the protein for the competition binding assay, the effective concentration of MDM2 (1–118) was each time ascertained by determining the apparent K_d towards 5'FAM-LTFEHYWAQLTS (P2, 10 nM). Competition assay was performed by contacting serial dilutions of tested compounds with 10 nM P2 at protein concentration yielding $f_0 = 0.8$. Fluorescence polarization was determined at 485 nm excitation and 535 nm emission 15 min after mixing all assay components. All tests were performed using Corning black 96-well NBS assay plates at room temperature.

1.4. NMR Experiments

Uniform ^{15}N isotope labeling was achieved by expression of the protein in the M9 minimal media containing $^{15}NH_4Cl$ as the sole nitrogen source. Final step of purification of MDM2/X for NMR consisted of gel filtration into the NMR buffer (50 mM phosphate buffer pH 7.4 containing 150 mM NaCl, 5 mM DTT). 10% (v/v) of D_2O was added to the samples to provide lock signal. Water suppression was carried out using the WATERGATE sequence.[2] All the spectra were recorded at 300K using a Bruker Avance 600 MHz spectrometer with the Cryo-Platform. ^{1}H-^{15}N heteronuclear correlations were obtained using the fast HSQC pulse sequence.[3] Assignment of the amide groups of MDM2 was obtained after Stoll et al.[4]

2. Synthetic procedures and analytical data

2.1 Synthetic procedure of α,ω-amino acids

The utilized amino acids were synthesized as previously reported;[5] The corresponding diamine (5.0 mmol) was dissolved in THF (30 mL) and then a solution of an anhydride (5.0 mmol) in THF (20 mL) was added dropwise during a 30 min period. The reaction mixture was further stirred for 1 h. The solvent was removed under vacuum and the resulting solid was washed with diethyl ether affording the targeted amino acids as white solids which were used without further purification.

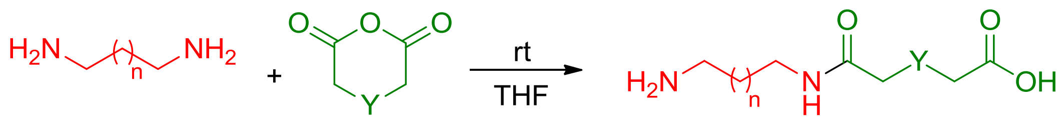

3 **4** **5**

n = 2-4,6,8,10

Y= O, S

Scheme S1. Reaction of unprotected diamines with cyclic anhydrides

2.2 Synthetic procedure and analytical data of the macrocycles

To a stirred solution of the α,ω-amino acid (1.5 mmol) in MeOH (5 mL), an aldehyde (1.0 mmol) was added and the reaction mixture was irradiated in microwave at 120 °C for 1 h. Afterwards, the reaction mixture was diluted by MeOH (10 mL), *tert*-butyl isocyanide was added and it was irradiated in microwave at 120 °C for an additional 1 h. The reaction progress was monitored by TLC (DCM/MeOH, 9:1). After completion of the reaction, the pure product was obtained by column chromatography (DCM/MeOH, 9:1).

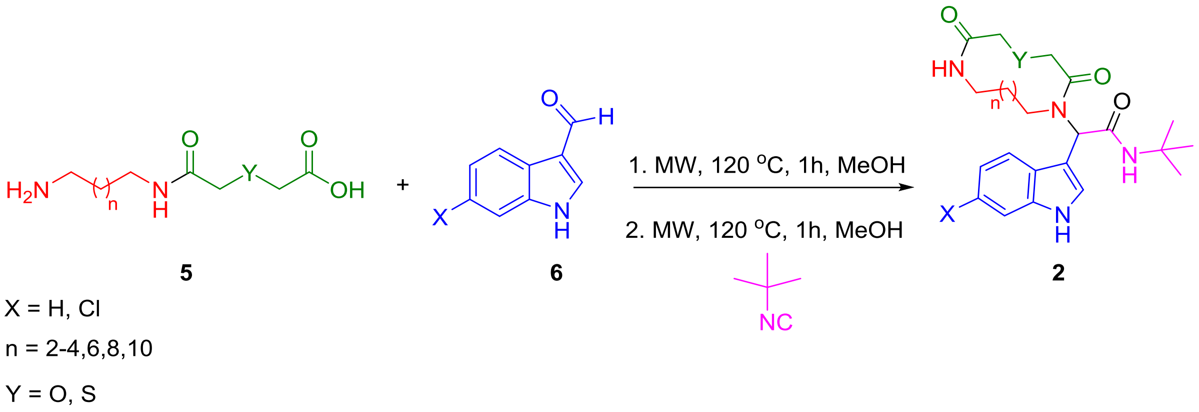

Scheme S2. Ugi macrocyclization in a one-pot fashion

N-(*tert*-Butyl)-2-(3,10-dioxo-1-oxa-4,9-diazacycloundecan-4-yl)-2-(1*H*-indol-3-yl) acetamide (**2a**)

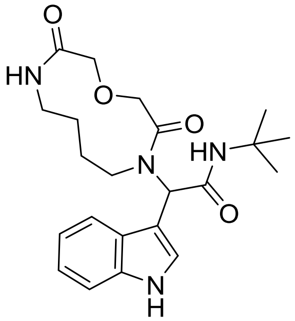

Yellow oil, 40% yield; ^{1}H NMR (500 MHz, Chloroform-*d*) δ 10.10 (s, 1H), 8.38 – 8.33 (m, 1H), 7.91 – 7.79 (m, 1H), 7.50 – 7.41 (m, 1H), 7.39 – 7.33 (m, 1H), 7.27 – 7.21 (m, 1H), 6.64 (s, 1H), 4.26 (s, 1H), 4.08 (s, 4H), 4.01 – 3.83 (m, 1H), 3.32 (dq, *J* = 6.3, 3.2, 2.7 Hz, 4H), 1.81 – 1.79 (m, 4H), 1.28 (s, 9H); ^{13}C NMR (126 MHz, Chloroform-*d*) δ 185.2, 170.0, 135.6, 124.3, 122.9, 121.9, 111.6, 74.8, 40.5, 29.7, 29.6, 29.5, 29.1, 27.4, 14.1. LC-MS (DAD/ESI): t_R = 5.25 min, calculated for $C_{22}H_{30}N_4O_4$ $[M+H]^+$: 415.23, found $[M+H]^+$: 415.23.

N-(*tert*-Butyl)-2-(3,11-dioxo-1-oxa-4,10-diazacyclododecan-4-yl)-2-(1*H*-indol-3-yl) acetamide (**2b**)

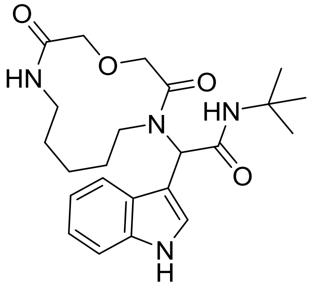

Yellow oil, 35% yield; ^{1}H NMR (500 MHz, Chloroform-*d*) δ 10.10 (s, 1H), 8.36 – 8.33 (m, 1H), 7.88 (d, *J* = 3.1 Hz, 1H), 7.50 – 7.42 (m, 2H), 7.38 – 7.33 (m, 2H), 7.11 (s, 1H), 6.75 (s, 1H), 6.40 (s, 1H), 4.98 (d, *J* = 10.5 Hz, 1H), 4.75 (s, 1H), 4.34 (dd, *J* = 44.7, 15.5 Hz, 2H), 4.21 (d, *J* = 12.8 Hz, 2H), 4.08 (d, *J* = 25.6 Hz, 5H), 4.03 – 3.93 (m, 1H), 3.89 (s, 1H), 3.66 – 3.54 (m, 2H), 3.48 – 3.40 (m, 5H), 2.69 (d, *J* = 14.1 Hz, 1H), 2.39 – 2.32 (m, 1H), 2.01 (s, 1H), 1.79 (s, 2H), 1.39 (s, 2H), 1.38 (s, 5H), 1.36 (d, *J* = 4.0 Hz, 10H), 1.28 (s, 7H), 0.94 – 0.82 (m, 5H); ^{13}C NMR (126 MHz, Chloroform-*d*) δ 185.2, 136.7, 135.6, 124.4, 124.3, 123.0, 122.9, 121.9, 119.6, 111.6, 74.4, 36.6, 29.7, 28.7, 28.6, 25.3, 19.7. LC-MS (DAD/ESI): t_R = 5.27 min, calculated for $C_{23}H_{32}N_4O_4$ $[M+H]^+$: 429.34, found $[M+H]^+$: 429.34.

N-(*tert*-Butyl)-2-(3,12-dioxo-1-oxa-4,11-diazacyclotridecan-4-yl)-2-(1*H*-indol-3-yl) acetamide (**2c**)

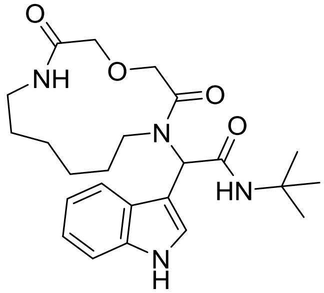

Yellow oil, 51% yield; ^{1}H NMR (500 MHz, Chloroform-*d*) δ 8.48 (s, 1H), 7.74 (d, *J* = 2.7 Hz, 1H), 7.46 (dd, *J* = 19.1, 8.0 Hz, 2H), 7.26 (ddd, *J* = 8.2, 7.1, 1.2 Hz, 1H), 7.19 – 7.14 (m, 1H), 5.32 (s, 2H), 4.40 – 4.30 (m, 2H), 4.12 (s, 1H), 4.09 – 4.06 (m, 2H), 3.81 (s, 1H), 3.44 – 3.38 (m, 2H), 3.33 (q, *J* = 6.6 Hz, 2H), 1.71 – 1.64 (m, 3H), 1.59 – 1.49 (m, 5H), 1.36 (s, 9H), 1.35 – 1.32 (m, 2H), 1.28 (s, 2H); ^{13}C NMR (126 MHz, Chloroform-*d*) δ 169.2, 168.6, 168.4, 135.6, 127.0, 125.9, 122.7, 120.3, 118.3, 111.4, 72.9, 71.3, 71.1, 70.7, 70.4, 68.6, 55.0, 52.1, 51.3, 46.2, 39.2, 38.9, 38.7, 38.5, 29.7, 29.3, 28.8, 28.6, 28.4, 26.1, 26.0, 25.8, 25.5, 25.3, 25.3. LC-MS (DAD/ESI): t_R = 5.26 min, calculated for $C_{24}H_{34}N_4O_4$ $[M+H]^+$: 443.33, found $[M+H]^+$: 443.33.

N-(*tert*-Butyl)-2-(3,14-dioxo-1-oxa-4,13-diazacyclopentadecan-4-yl)-2-(1*H*-indol-3-yl) acetamide (**2d**)

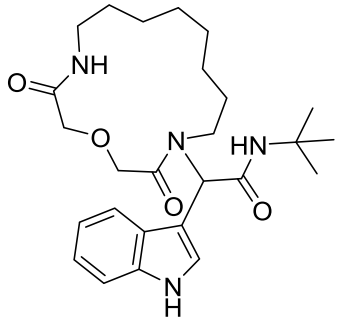

Yellow oil, 33% yield; ^{1}H NMR (500 MHz, Chloroform-*d*) δ 10.07 (s, 4H), 8.34 (ddd, *J* = 7.0, 3.3, 2.0 Hz, 5H), 8.07 (d, *J* = 2.1 Hz, 1H), 7.86 (s, 5H), 7.63 (d, *J* = 2.5 Hz, 1H), 7.53 (d, *J* = 8.0 Hz, 1H), 7.50 – 7.45 (m, 6H), 7.36 – 7.32 (m, 9H), 6.45 (t, *J* = 5.9 Hz, 1H), 6.18 (s, 1H), 6.05 (s, 1H), 4.39 – 4.28 (m, 2H), 4.20 (d, *J* = 2.2 Hz, 2H), 4.16 (s, 2H), 4.12 – 4.05 (m, 4H), 3.80 (d, *J* = 3.5 Hz, 2H), 3.64 (t, *J* = 6.8 Hz, 1H), 3.41 – 3.37 (m, 1H), 3.35 (d, *J* = 9.5 Hz, 2H), 3.30 (qd, *J* = 6.9, 4.6 Hz, 3H), 3.24 – 3.16 (m, 1H), 3.13 – 3.06 (m, 1H), 1.78 (q, *J* = 7.2 Hz, 1H), 1.62 – 1.50 (m, 6H), 1.47 (s, 2H), 1.41 (s, 2H), 1.37 (s, 9H), 1.36 (s, 9H), 1.34 – 1.15 (m, 16H), 1.06 (s, 4H); ^{13}C NMR (126 MHz, Chloroform-*d*) δ 23.0, 23.5, 23.7, 25.2, 25.4, 25.7, 27.1, 27.3, 28.6, 28.8, 28.9, 29.1, 29.4, 30.9, 37.7, 38.3, 51.5, 68.6, 70.0, 70.8, 70.9, 111.5, 111.6, 118.4, 119.5, 120.4,

121.9, 122.8, 122.9, 124.3, 124.4, 125.7, 135.6, 136.8, 185.2. LC-MS (DAD/ESI): t_R = 5.24 min, calculated for $C_{26}H_{38}N_4O_4$ $[M+H]^+$: 471.27, found $[M+H]^+$: 471.27.

N-(*tert*-Butyl)-2-(3,16-dioxo-1-oxa-4,15-diazacycloheptadecan-4-yl)-2-(1*H*-indol-3-yl) acetamide (**2e**)

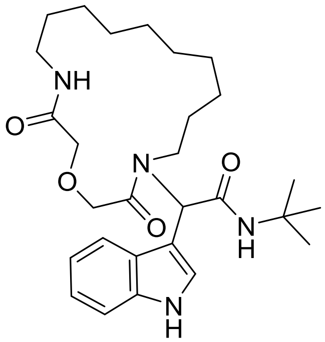

Yellow oil, 31% yield; ^{1}H NMR (500 MHz, Chloroform-*d*) δ 8.64 – 8.57 (m, 1H), 7.63 (d, *J* = 2.6 Hz, 1H), 7.52 (d, *J* = 8.0 Hz, 1H), 7.44 (d, *J* = 8.2 Hz, 1H), 7.26 (t, *J* = 7.3 Hz, 1H), 7.18 (dt, *J* = 15.0, 7.3 Hz, 1H), 6.27 (s, 1H), 6.02 (s, 1H), 4.39 – 4.28 (m, 2H), 4.19 (d, *J* = 3.0 Hz, 1H), 4.08 (s, 2H), 3.44 – 3.37 (m, 3H), 3.39 – 3.29 (m, 2H), 3.24 (ddd, *J* = 15.9, 11.3, 4.8 Hz, 1H), 3.15 (ddd, *J* = 15.6, 10.9, 5.2 Hz, 1H), 1.60 (td, *J* = 9.5, 7.7, 4.9 Hz, 2H), 1.56 – 1.47 (m, 2H), 1.43 – 1.37 (m, 5H), 1.36 (s, 10H), 1.31 (d, *J* = 9.1 Hz, 4H), 1.26 – 1.21 (m, 2H), 1.16 – 1.08 (m, 2H), 1.07 (d, *J* = 6.4 Hz, 1H), 0.96 (ddq, *J* = 23.5, 11.4, 6.4, 5.9 Hz, 2H). ^{13}C NMR (126 MHz, Chloroform-*d*) δ 169.2, 169.1, 168.7, 168.3, 135.7, 126.9, 125.7, 122.8, 120.4, 118.4, 111.4, 109.2, 71.1, 70.9, 69.4, 54.7, 51.4, 44.9, 38.4, 38.2, 28.7, 28.6, 28.5, 28.0, 27.7, 27.1, 26.95, 26.8, 26.6326.40, 26.10, 25.7, 25.3, 25.1. LC-MS (DAD/ESI): t_R = 5.84 min, calculated for $C_{28}H_{42}N_4O_4$ $[M+H]^+$: 499.39, found $[M+H]^+$: 499.39.

N-(*tert*-Butyl)-2-(3,18-dioxo-1-oxa-4,17-diazacyclononadecan-4-yl)-2-(1*H*-indol-3-yl) acetamide (**2f**)

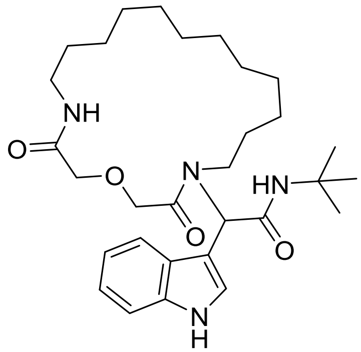

Yellow oil, 59% yield; ^{1}H NMR (500 MHz, Chloroform-*d*) δ 10.09 (s, 1H), 8.43 – 8.25 (m, 1H), 7.86 (d, *J* = 3.1 Hz, 1H), 7.53 – 7.47 (m, 1H), 7.47 – 7.42 (m, 1H), 7.37 – 7.35 (m, 1H), 7.35 – 7.33 (m, 1H), 7.18 (td, *J* = 15.8, 7.9 Hz, 1H), 4.42 – 4.28 (m, 1H), 4.20 (d, *J* = 4.3 Hz, 1H), 4.09 (s, 1H), 3.81 (s, 1H), 3.44 – 3.36 (m, 1H), 3.35 – 3.12 (m, 2H), 1.61 – 1.55 (m, 1H), 1.55 – 1.48 (m, 1H), 1.45 (s, 1H), 1.41 (s, 3H), 1.37 (d, *J* = 3.0 Hz, 9H), 1.33 (s, 1H), 1.32 (d, *J* = 2.9 Hz, 1H), 1.28 (s, 3H), 1.19 (s, 2H), 1.12 (s, 1H), 0.98 (s, 2H); ^{13}C NMR (126 MHz, Chloroform-*d*) δ 185.1,168.9, 135.4, 124.3, 12.0, 122.7, 121.9, 118.4, 111.5, 71.6,71.1, 70.2, 68.5, 44.7, 38.7, 30.9, 29.5, 28.9, 28.8, 28.6, 27.6, 27.2, 27.0, 26.4, 25.6, 25.3. LC-MS (DAD/ESI): t_R = 5.30 min, calculated for $C_{30}H_{46}N_4O_4$ $[M+H]^+$: 527.34, found $[M+H]^+$: 527.34.

N-(*tert*-Butyl)-2-(6-chloro-1*H*-indol-3-yl)-2-(3,12-dioxo-1-oxa-4,11-diazacyclotridecan-4-yl)acetamide (**2g**)

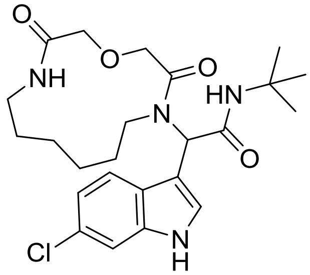

Yellow solid, 35% yield; ^{1}H NMR (500 MHz, Chloroform-*d*) 9.32 (s, 1H), 7.68 (s, 1H), 7.44 (s, 1H), 7.38 (d, J = 10 Hz, 1H), 7.29 (s, 1H), 7.11-7.09 (m, 1H), 6.57-6.55 (m, 1H), 6.20 (s, 1H), 6.10 (s, 1H), 4.32-4.31 (m, 2H), 4.04-4.03 (m, 2H), 3.42-3.11 (m, 6H), 1.52-1.37 (m, 4H), 1.34 (s, 9H), 1.22-1.14 (m, 4H); ^{13}C NMR (126 MHz, CDCl3) δ 169.1, 168.7, 168.5, 136.3, 128.5, 126.8, 125.6, 121.0, 119.2, 111.7, 108.9, 73.0, 70.54, 70.5, 55.1, 51.5, 46.3, 39.3, 28.6, 28.4, 25.9, 25.5, 25.2. LC-MS (DAD/ESI): t_R = 3.81 min, calculated for $C_{24}H_{33}ClN_4O_4$ $[M+Na]^+$: 499.22, found $[M+Na]^+$: 499.29.

N-(*tert*-Butyl)-2-(6-chloro-1*H*-indol-3-yl)-2-(3,14-dioxo-1-oxa-4,13-diazacyclopentadecan-4-yl)acetamide (**2h**)

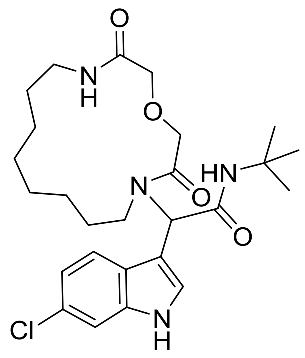

Yellow solid, 25% yield; ^{1}H NMR (500 MHz, Chloroform-*d*); ^{1}H NMR (500 MHz, CDCl3) δ 8.48 (s, 1H), 7.70 (d, J = 2.5 Hz, 1H), 7.46 – 7.38 (m, 2H), 7.14 (dd, J = 8.5, 1.8 Hz, 1H), 6.51 – 6.39 (m, 1H), 6.21 (s, 1H), 6.06 (s, 1H), 4.44 – 4.27 (m, 2H), 4.17 (s, 2H), 3.44 – 3.32 (m, 2H), 3.26 –3.15 (m, 1H), 3.13 – 3.01 (m, 1H), 1.69 – 1.56 (m, 7H), 1.36 (s, 11H), 1.26 – 1.21 (m, 3H); ^{13}C NMR (126 MHz, Chloroform-*d*) δ 168.9, 168.8, 168.6, 136.1, 128.8, 126.6, 125.5, 121.2, 119.4, 111.4, 109.5, 70.9, 70.1, 54.4, 51.5, 45.6, 37.8, 28.6, 28.2, 27.2, 25.5, 25.2, 23.7, 23.1; HRMS (ESI) m/z calculated for $C_{26}H_{38}ClN_4O_4$ $[M+H]^+$: 504.2576, found $[M+H]^+$: 505.2576.

N-(*tert*-Butyl)-2-(6-chloro-1*H*-indol-3-yl)-2-(3,16-dioxo-1-oxa-4,15-diazacycloheptadecan-4-yl)acetamide (**2i**)

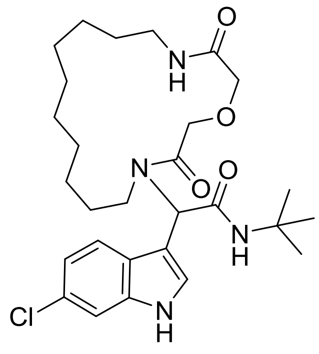

Yellow oil, 51% yield; ^{1}H NMR (500 MHz, Chloroform-*d*) *δ* 10.04 (s, 1H), 8.30 (d, *J* = 12.4 Hz, 1H), 8.06 (d, *J* = 2.0 Hz, 1H), 7.46 – 7.40 (m, 1H), 7.30 (dd, *J* = 5.6, 1.8 Hz, 1H), 5.80 (s, 1H), 5.32 (s, 1H), 4.39 – 4.29 (m, 1H), 4.19 (d, *J* = 6.0 Hz, 1H), 4.09 (s, 1H), 3.81 (d, *J* = 6.7 Hz, 1H), 3.51 (s, 2H), 3.36 – 3.28 (m, 1H), 1.41 (s, 10H), 1.37 (s, 12H), 1.33 – 1.25 (m, 4H); ^{13}C NMR (126 MHz, Chloroform-*d*) *δ* 169.2, 168.8, 168.4, 136.1, 126.4, 123.5, 122.8, 121.2, 119.3, 111.7, 111.5, 71.0, 70.9, 54.6, 51.58, 44.98, 38.2, 28.7, 28.6, 28.0, 27.7, 27.1, 26.9, 26.8, 26.6, 26.3, 26.0, 25.7, 25.3, 25.1. LC-MS

(DAD/ESI): t_R = 5.52 min, calculated for $C_{28}H_{41}ClN_4O_4$ $[M+H]^+$: 533.31, found $[M+H]^+$: 533.31.

N-(*tert*-Butyl)-2-(6-chloro-1*H*-indol-3-yl)-2-(3,18-dioxo-1-oxa-4,17-diazacyclononadecan-4-yl)acetamide (**2j**)

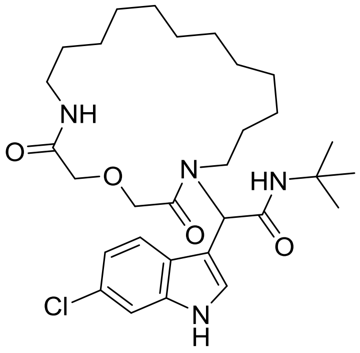

Yellow oil, 60% yield; ^{1}H NMR (500 MHz, Chloroform-*d*) δ 8.99 (s, 1H), 7.62 (d, *J* = 2.4 Hz, 1H), 7.44 – 7.41 (m, 1H), 7.11 (dd, *J* = 8.5, 1.7 Hz, 1H), 6.22 (s, 1H), 6.09 (s, 1H), 4.38 – 4.26 (m, 2H), 4.21 – 4.14 (m, 2H), 3.37 (t, *J* = 6.2 Hz, 2H), 3.32 – 3.25 (m, 2H), 1.61 – 1.55 (m, 3H), 1.50 (q, *J* = 8.6, 7.9 Hz, 2H), 1.36 (d, *J* = 2.6 Hz, 5H), 1.35 (s, 9H), 1.32 (d, *J* = 8.1 Hz, 5H), 1.28 – 1.25 (m, 4H); ^{13}C NMR (126 MHz, Chloroform-*d*) δ 169.2, 169.1, 168.9, 136.2, 128.6, 126.5, 125.5, 121.1, 119.3, 111.5, 109.4, 71.2, 70.1, 54.5, 51.5, 44.7, 38.7, 28.8, 28.6, 27.5, 27.5, 27.2, 27.2, 27.1, 27.0, 26.4, 26.0, 25.6, 25.3. LC-MS (DAD/ESI): t_R = 5.53min, calculated for $C_{30}H_{45}ClN_4O_4$ $[M+H]^+$: 561.36 found $[M+H]^+$: 561.36.

N-(*tert*-Butyl)-2-(6-chloro-1*H*-indol-3-yl)-2-(3,10-dioxo-1-thia-4,9-diazacycloundecan-4-yl)acetamide (**2k**)

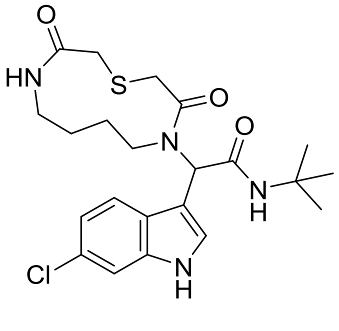

Yellow oil, 29% yield; ^{1}H NMR (500 MHz, Chloroform-*d*) δ 9.36 (s, 1H), 8.23 (d, *J* = 8.5 Hz, 1H), 7.89 (d, *J* = 1.9 Hz, 1H), 7.54 – 7.51 (m, 2H), 7.50 (dd, *J* = 4.7, 1.8 Hz, 2H), 7.48 – 7.37 (m, 2H), 7.27 – 7.25 (m, 2H), 7.22 – 7.16 (m, 3H), 6.03 (s, 1H), 5.97 (d, *J* = 9.3 Hz, 1H), 3.49 (d, *J* = 7.7 Hz, 1H), 3.43 – 3.40 (m, 1H), 3.37 – 3.33 (m, 2H), 3.30 –

3.25 (m, 4H), 1.78 (p, *J* = 2.4 Hz, 4H), 1.31 (s, 9H); ^{13}C NMR (126 MHz, CDCl3) *δ* 169.7, 169.5, 168.8, 136.6, 136.2, 128.8, 126.2, 125.5, 123.4, 122.7, 121.3, 118.8, 112.2, 111.9, 111.9, 77.2, 77.0, 76.7, 51.8, 41.2, 41.0, 40.6, 38.3, 36.4, 34.7, 31.8, 30.9, 29.7, 29.6, 28.7, 28.6, 28.4, 28.3, 27.3, 27.3, 26.0, 8.6. LC-MS (DAD/ESI): t_R = 7.10 min, calculated for $C_{22}H_{29}ClN_4O_3S$ $[M+H]^+$: 465.23, found $[M+H]^+$: 465.23.

N-(*tert*-Butyl)-2-(6-chloro-1*H*-indol-3-yl)-2-(3,11-dioxo-1-thia-4,10-diazacyclododecan-4-yl)acetamide (**2l**)

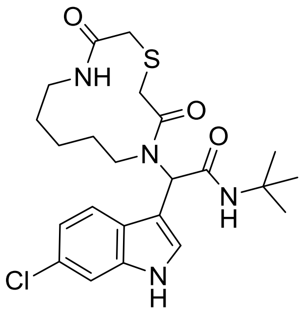

Yellow oil, 35% yield; ^{1}H NMR (500 MHz, Chloroform-*d*) *δ* 8.07 (d, *J* = 2.0 Hz, 1H), 7.87 (d, *J* = 3.0 Hz, 1H), 7.47 (d, *J* = 1.9 Hz, 1H), 7.32 (d, *J* = 1.8 Hz, 1H), 7.30 (d, *J* = 1.9 Hz, 1H), 3.78 (d, *J* = 9.5 Hz, 1H), 3.35 (d, *J* = 4.6 Hz, 1H), 3.34 – 3.29 (m, 1H), 1.42 (s, 9H), 1.39 (s, 1H), 1.37 (s, 9H), 1.35 (d, *J* = 1.4 Hz, 1H); ^{13}C NMR (126 MHz, $CDCl_3$) *δ* 168.9, 128.6, 126.5, 125.5, 121.1, 119.2, 111.5, 77.2, 77.0, 76.7, 52.1, 51.5, 40.4, 38.2, 37.1, 33.1, 31.1, 30.3, 29.7, 28.7, 28.5, 28.3, 27.9. LC-MS (DAD/ESI): t_R = 3.85 min, calculated for $C_{23}H_{31}ClN_4O_3S$ $[M+H]^+$: 479.26, found $[M+H]^+$: 479.26.

N-(*tert*-Butyl)-2-(6-chloro-1*H*-indol-3-yl)-2-(3,12-dioxo-1-thia-4,11-diazacyclotridecan-4-yl)acetamide (**2m**)

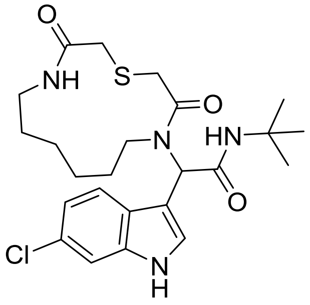

Pale-white solid, 39% yield; ^{1}H NMR (500 MHz, Chloroform-*d*) δ 9.36 (s, 1H), 7.61 (s, 2H), 7.47 – 7.33 (m, 2H), 7.25 (d, *J* = 26.7, 2H), 7.09 (d, *J* = 7.8, 1H), 6.11 (s, 1H), 5.92 (s, 1H), 3.79 – 3.00 (m, 12H), 2.01 (d, *J* = 36.1, 2H), 1.74 – 0.99 (m, 25H); ^{13}C NMR (126 MHz, Chloroform-*d*) δ 169.1, 168.6, 136.5, 128.7, 126.9, 125.8, 121.1, 119.4, 111.9, 109.5, 55.6, 51.7, 45.6, 39.6, 39.2, 36.2, 35.1, 31.1, 28.7, 27.2, 27.1, 27.0, 24.9, 24.3, 24.2. LC-MS (DAD/ESI): t_R = 4.11 min, calculated for $C_{24}H_{33}ClN_4O_3S$ $[M-H]^-$: 491.20, found $[M-H]^-$: 491.23.

N-(*tert*-Butyl)-2-(6-chloro-1*H*-indol-3-yl)-2-(3,14-dioxo-1-thia-4,13-diazacyclopentadecan-4-yl)acetamide (**2n**)

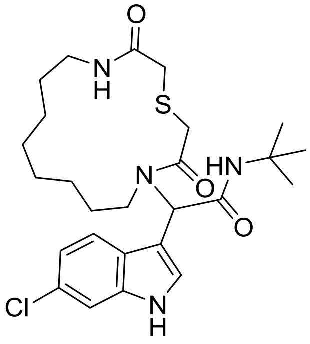

Yellow oil, 39% yield; ^{1}H NMR (500 MHz, Chloroform-*d*) δ 8.77 – 8.74 (m, 1H), 8.24 (d, *J* = 8.5 Hz, 1H), 7.86 (s, 1H), 7.62 (d, *J* = 2.4 Hz, 1H), 7.48 (d, *J* = 1.9 Hz, 1H), 7.43 (d, *J* = 1.7 Hz, 1H), 6.21 (s, 1H), 6.04 (s, 1H), 3.91 – 3.75 (m, 2H), 3.69 – 3.60 (m, 2H), 3.32 – 3.26 (m, 2H), 3.23 – 3.13 (m, 2H), 1.56 (tdd, *J* = 7.5, 6.3, 5.5, 3.2 Hz, 5H), 1.41 (q, *J* = 2.6, 2.2 Hz, 2H), 1.35 (d, *J* = 2.3 Hz, 9H), 1.32 (t, *J* = 4.5 Hz, 5H); ^{13}C NMR (126 MHz, Chloroform-*d*) δ 168.9, 168.5, 168.1, 136.2, 126.5, 123.5, 122.8, 121.1, 119.5, 111.8, 111.5, 54.7, 51.5, 45.9, 38.6, 36.4, 35.8, 29.7, 28.6, 28.4, 28.2, 27.8, 25.9, 23.2. LC-MS (DAD/ESI): t_R = 5.71 min, calculated for $C_{26}H_{37}ClN_4O_3S$ $[M+H]^+$: 521.30, found $[M+H]^+$: 521.30.

N-(*tert*-Butyl)-2-(6-chloro-1*H*-indol-3-yl)-2-(3,16-dioxo-1-thia-4,15-diazacycloheptadecan-4-yl)acetamide (**2o**)

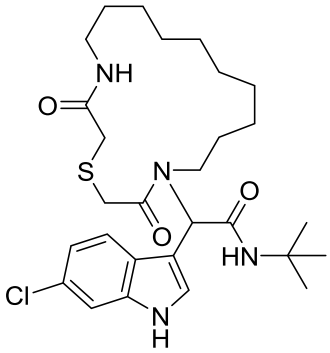

Yellow oil, 41% yield; ^{1}H NMR (500 MHz, Chloroform-*d*) δ 8.93 (s, 1H), 8.42 (s, 1H), 8.19 (dd, *J* = 41.2, 8.5 Hz, 1H), 7.60 (d, *J* = 2.4 Hz, 1H), 7.51 – 7.42 (m, 2H), 6.19 (d, *J* = 10.8 Hz, 1H), 6.08 (d, *J* = 7.3 Hz, 1H), 3.66 – 3.60 (m, 2H), 3.57 (d, *J* = 4.6 Hz, 1H), 3.50 (q, *J* = 7.1 Hz, 1H), 3.31 – 3.25 (m, 3H), 3.25 – 3.17 (m, 2H), 1.55 (dt, *J* = 19.5, 6.3 Hz, 7H), 1.35 (s, 9H), 1.31 – 1.27 (m, 7H); ^{13}C NMR (126 MHz, Chloroform-*d*) δ 169.6, 168.6, 168.4, 136.2, 128.6, 126.5, 123.4, 121.0, 119.4, 111.9, 111.5, 55.3, 52.6, 51.5, 46.2, 39.2, 38.0, 29.3, 29.1, 28.8, 28.6, 28.3, 26.6, 26.4, 25.7, 25.0. LC-MS (DAD/ESI): t_R = 5.75 min, calculated for $C_{28}H_{41}ClN_4O_3S$ $[M+H]^+$: 549.28, found $[M+H]^+$: 549.28.

N-(*tert*-Butyl)-2-(6-chloro-1*H*-indol-3-yl)-2-(3,18-dioxo-1-thia-4,17-diazacyclononadecan-4-yl)acetamide (**2p**)

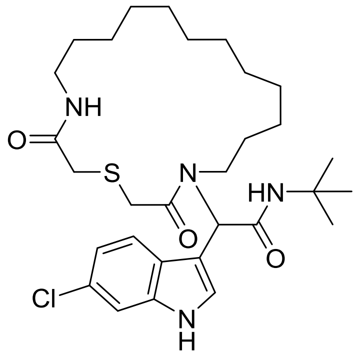

Yellow oil, 52% yield; ^{1}H NMR (500 MHz, Chloroform-*d*) δ 10.03 (s, 1H), 8.31 (d, *J* = 12.4 Hz, 1H), 8.24 (d, *J* = 8.5 Hz, 1H), 8.07 (d, *J* = 2.1 Hz, 1H), 7.84 (d, *J* = 2.9 Hz, 1H), 7.47 – 7.41 (m, 2H), 5.92 – 5.72 (m, 1H), 5.34 (d, *J* = 13.6 Hz, 1H), 3.60 – 3.49 (m, 2H), 3.39 – 3.25 (m, 6H), 1.56 (dt, *J* = 11.9, 6.1 Hz, 2H), 1.41 (s, 9H), 1.37 (s, 9H), 1.34 (d, *J* = 6.3 Hz, 7H), 1.28 – 1.24 (m, 3H); ^{13}C NMR (126 MHz, Chloroform-*d*) δ 185.0, 169.0, 137.2, 136.1, 130.1, 126.3, 123.6, 122.8, 119.4, 111.7, 39.8, 39.5, 36.8, 35.71, 34.09, 30.9, 28.9, 28.8, 28.6, 28.6, 28.4, 27.5, 27.3, 27.2, 27.1, 27.0, 26.8, 25.8, 25.6, 25.5. LC-MS (DAD/ESI): t_R = 5.81 min, calculated for $C_{30}H_{45}ClN_4O_3S$ $[M+H]^+$: 577.37, found $[M+H]^+$: 577.37.

3. Exemplary copies of NMR and MS data of novel compounds

N-(*tert*-Butyl)-2-(3,12-dioxo-1-oxa-4,11-diazacyclotridecan-4-yl)-2-(1*H*-indol-3-yl)acetamide (**2c**)

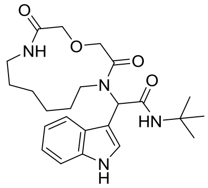

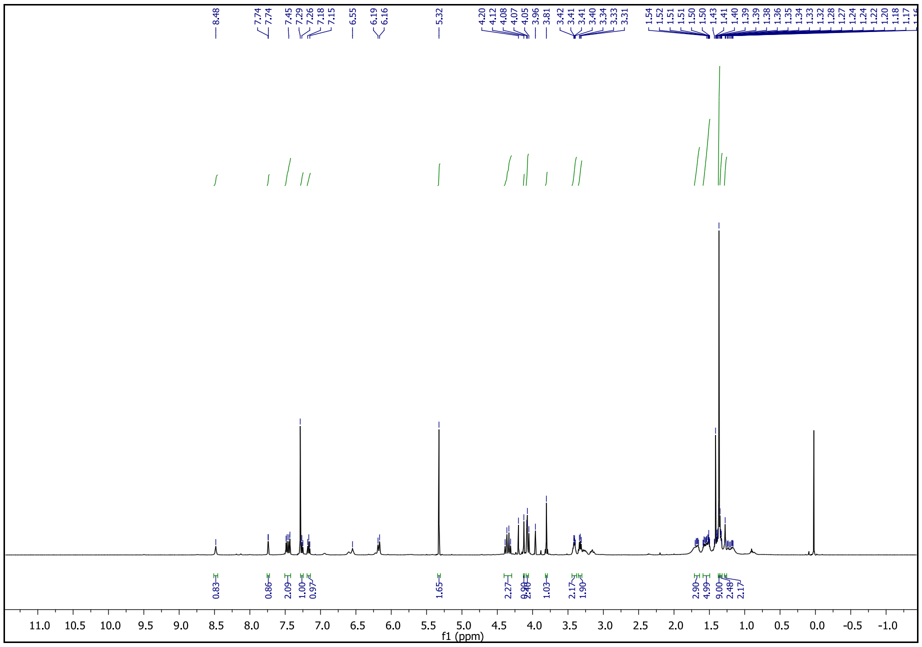

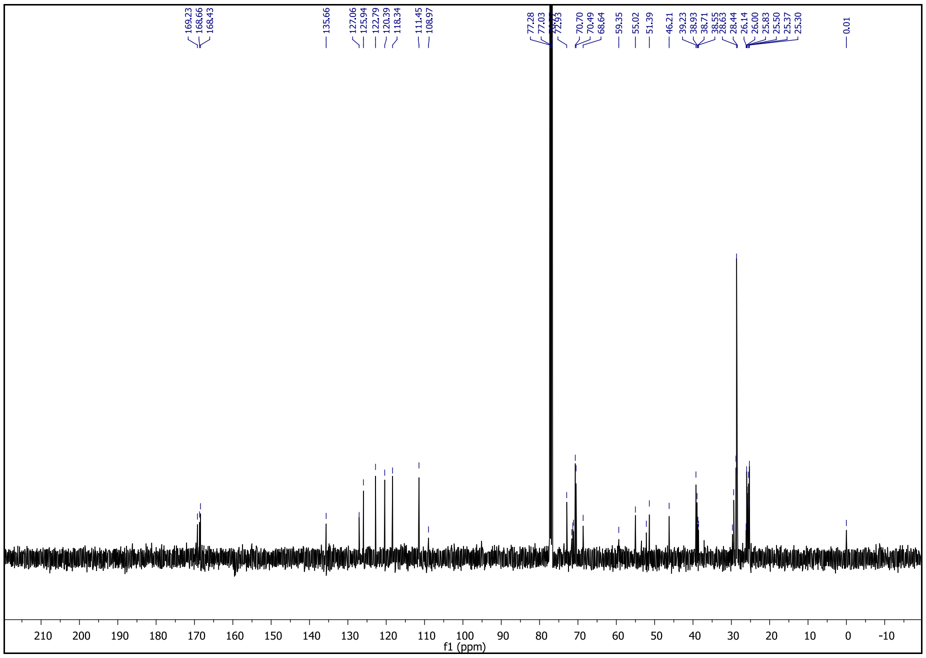

^{1}H and ^{13}C NMR spectra of compound ***2c*** *in $CDCl_3$*

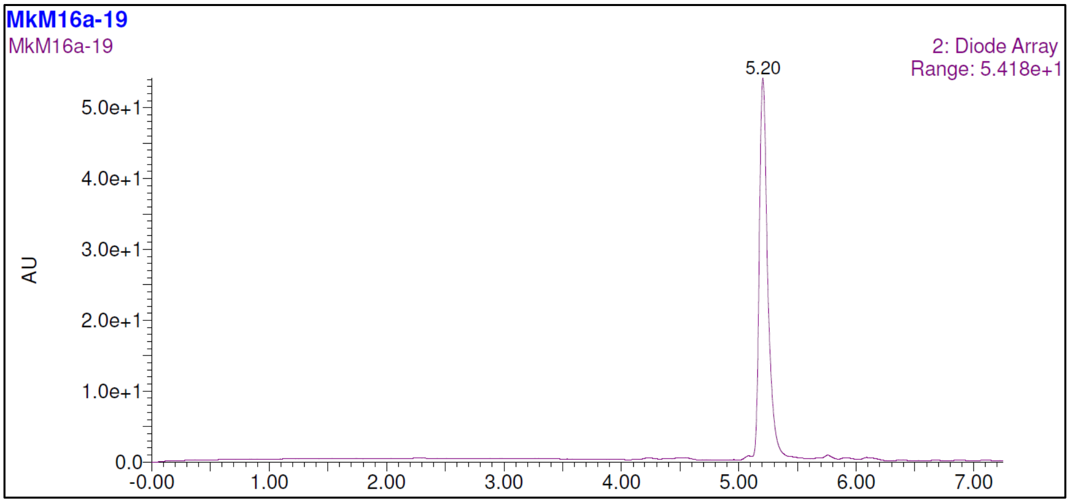

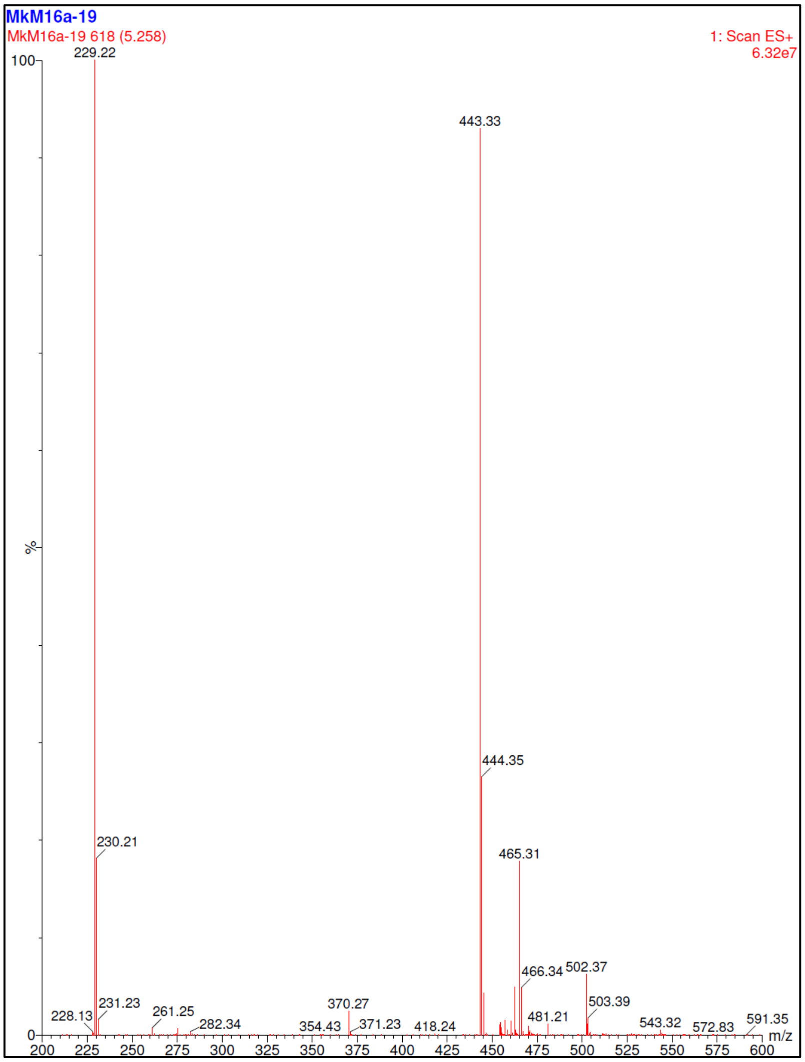

*Chromatogram and mass spectrum of compound **2c***

N-(*tert*-Butyl)-2-(6-chloro-1*H*-indol-3-yl)-2-(3,12-dioxo-1-oxa-4,11-diazacyclotridecan-4-yl)acetamide (**2g**)

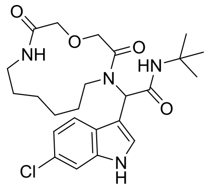

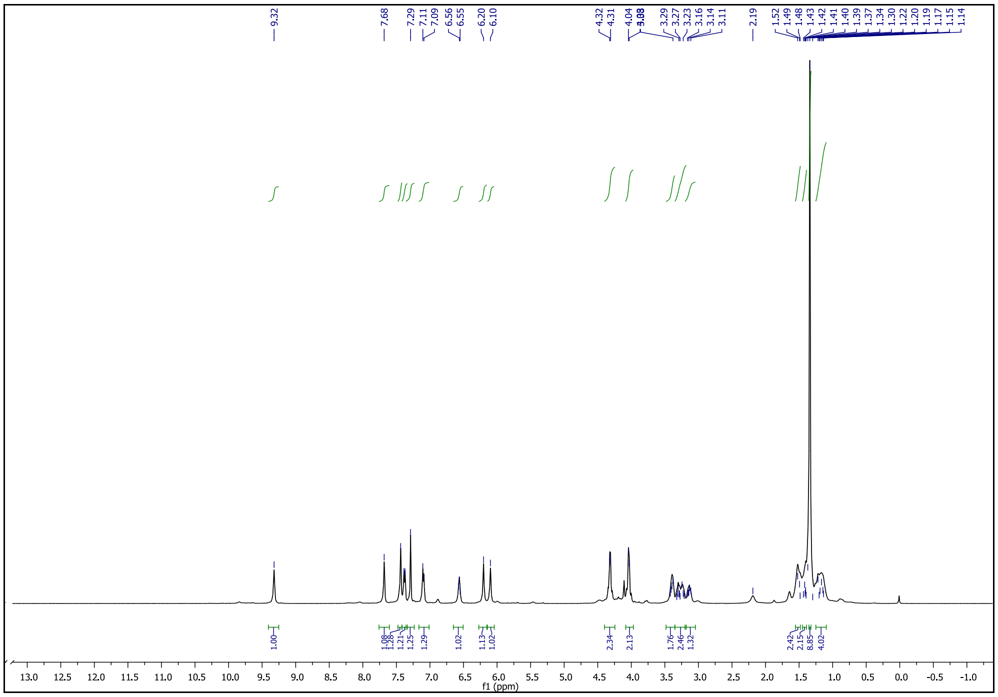

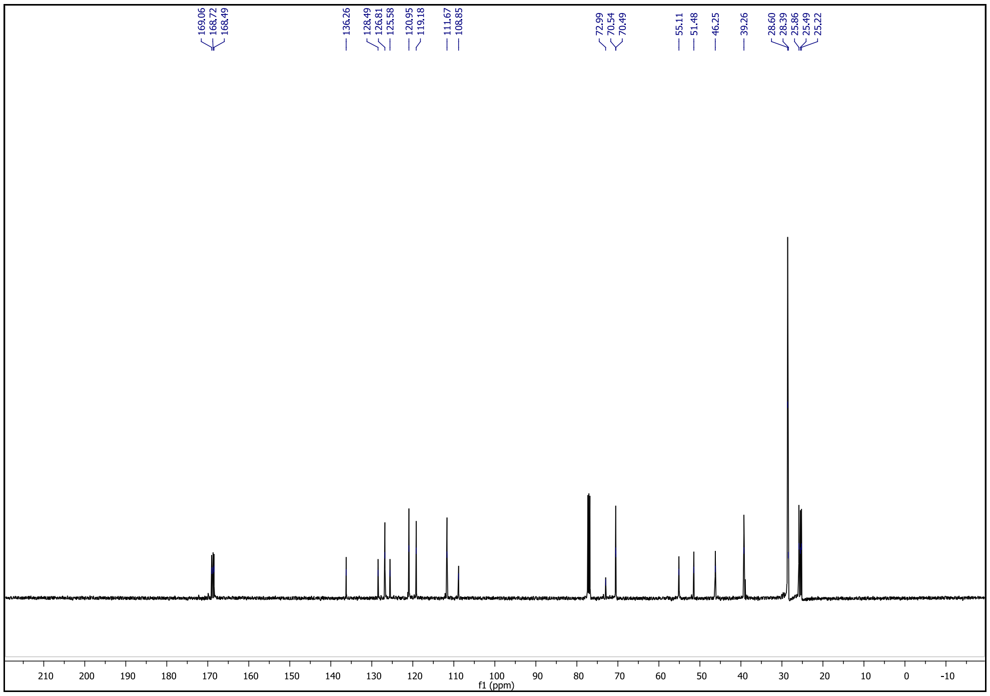

^{1}H and ^{13}C NMR spectra of compound ***2g*** *in $CDCl_3$*

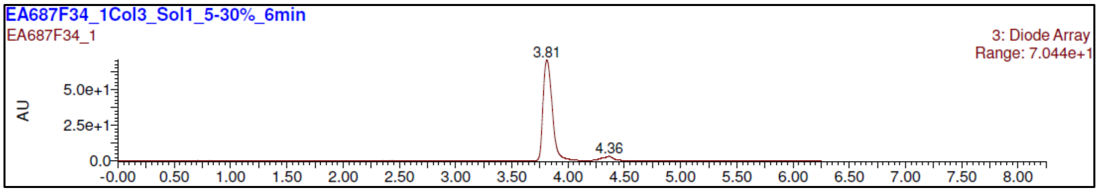

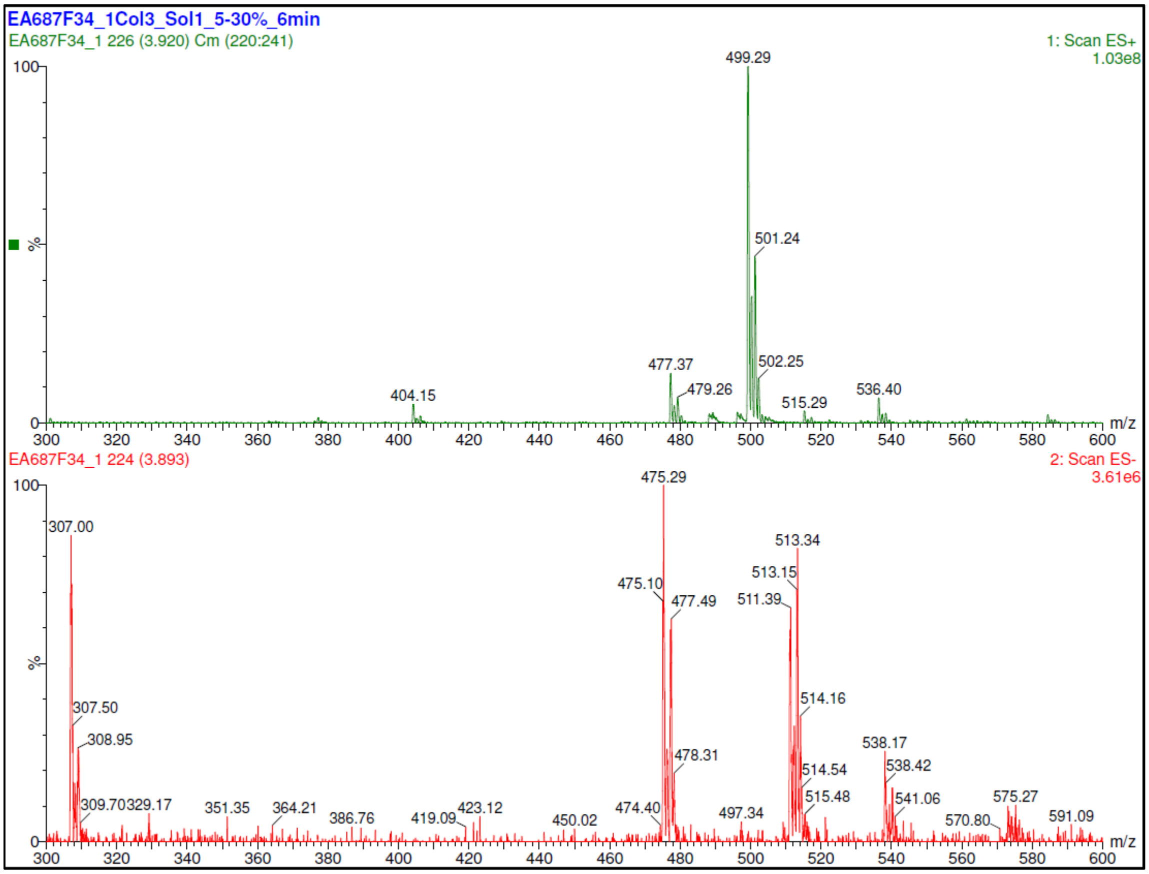

*Chromatogram and mass spectrum of compound **2g***

N-(*tert*-Butyl)-2-(6-chloro-1*H*-indol-3-yl)-2-(3,12-dioxo-1-thia-4,11-diazacyclotridecan-4-yl)acetamide (**2m**)

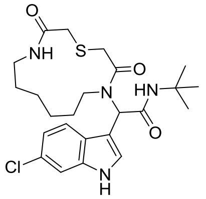

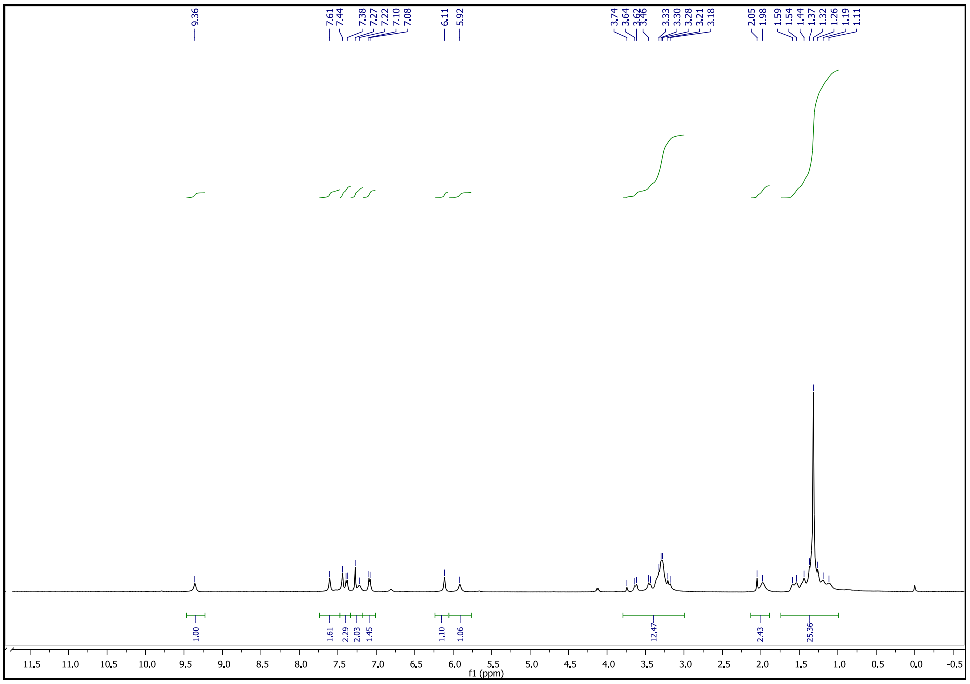

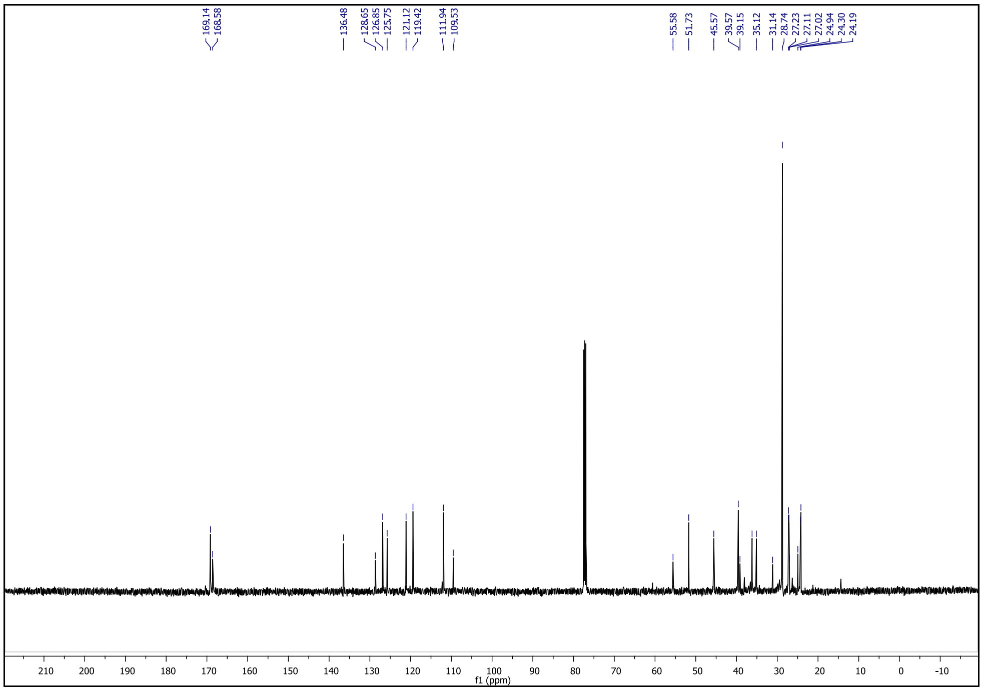

*^{1}H and ^{13}C NMR spectra of compound **2m** in $CDCl_3$*

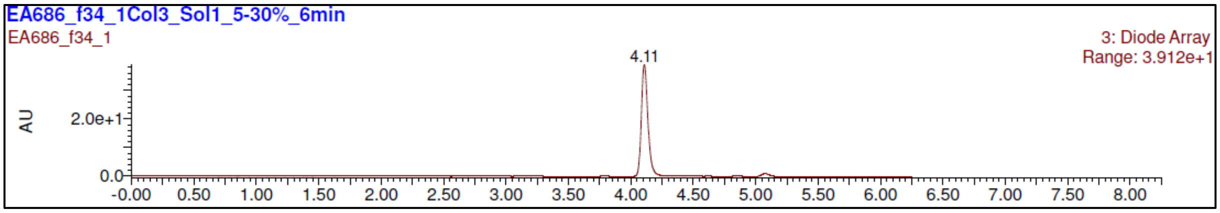

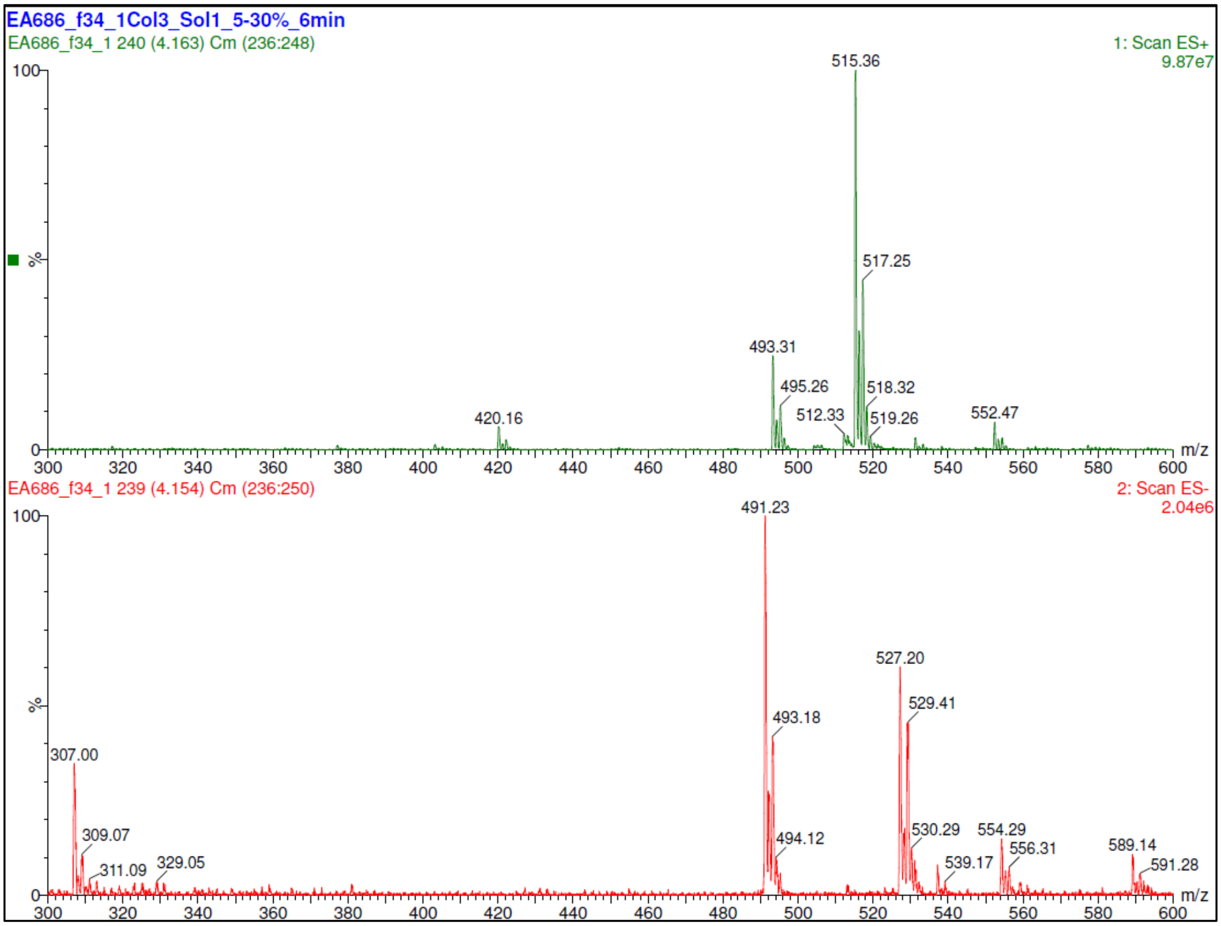

*Chromatogram and mass spectrum of compound **2m***

4. Fluorescence polarization binding assays

Table S1. Fluorescence polarization binding assays and the determination of the K_i values

Entry	Name	Structure	K_i MDM2 [µM]	Plot (MDM2)
1	**2h**	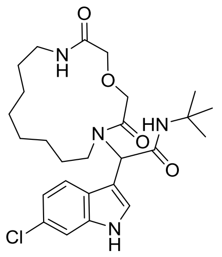	2.25	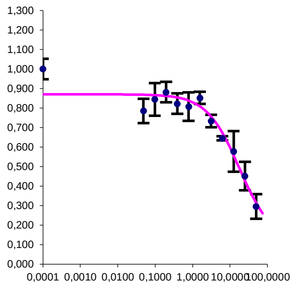
2	**2i**	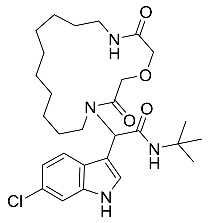	5.49	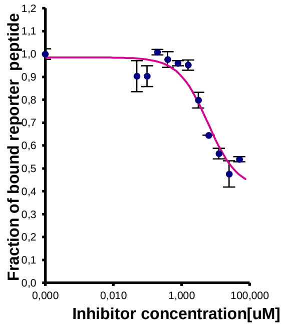
3	**2n**	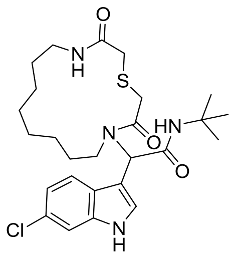	316.2	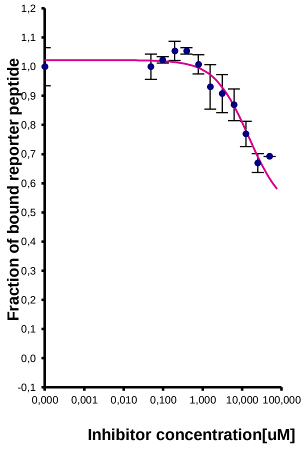

5. Titration of ^{15}N-labeled MDM2 with inhibitors

For each compound the two-dimensional ^{1}H-^{15}N correlated NMR spectrum was recorded at 7–9 different ligand/protein ratios from 0 to 5. The samples were prepared by adding small amounts of a 50 mM ligand stock solution in DMSO to the protein solution (0.20 mL) containing the ^{15}N labeled Mdm2 fragment at a concentration of 0.20–0.35 mM. The acquisition parameters for each HSQC spectrum was as follows: size of the FID F2: 2048, F1: 100, and number of scans: 10. Spectra were visualized using *TopSpin 4.0.2*. For the determination of dissociation constants (K_D), nonlinear fits of chemical shifts of single residue versus ligand ratio were performed using the program *OriginPro* (version 9.1) according to the following equation:[6-8]

$$\Delta_{obs} = \delta_M \frac{([P]_0 + [L]_0 + K_D) - \sqrt{([P]_0 + [L]_0 + K_D)^2 - 4[P]_0[L]_0}}{2[P]_0}$$

Where

$[L]_0$ – ligand concentration

$[P]_0$ – protein concentration

δ_{obs} – observed chemical shift perturbation normalized according to the Pythagoras formula with ^{15}N weighting factor of 0.2.

δ_M – chemical shift for single residue obtained with maximum ligand concentration

K_D – dissociation constants

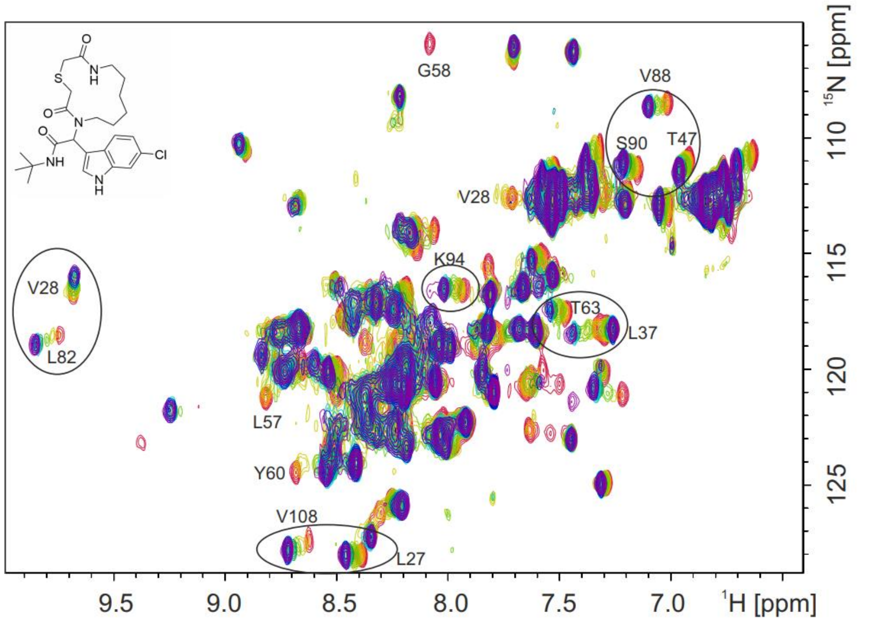

Figure S1. Overlay of ^{1}H-^{15}N-HSQC spectra of the reference MDM2 (red) and the titration steps with **2g** inhibitor. MDM2/**2g** ratios 8:0.5 (orange), 4:0.5 (yellow), 4:0.75 (light green), 4:2 (dark green), 4:3 (light blue), 1:1 (blue), 1:2 (purple). Examples of most perturbed residues are labeled on the spectrum.

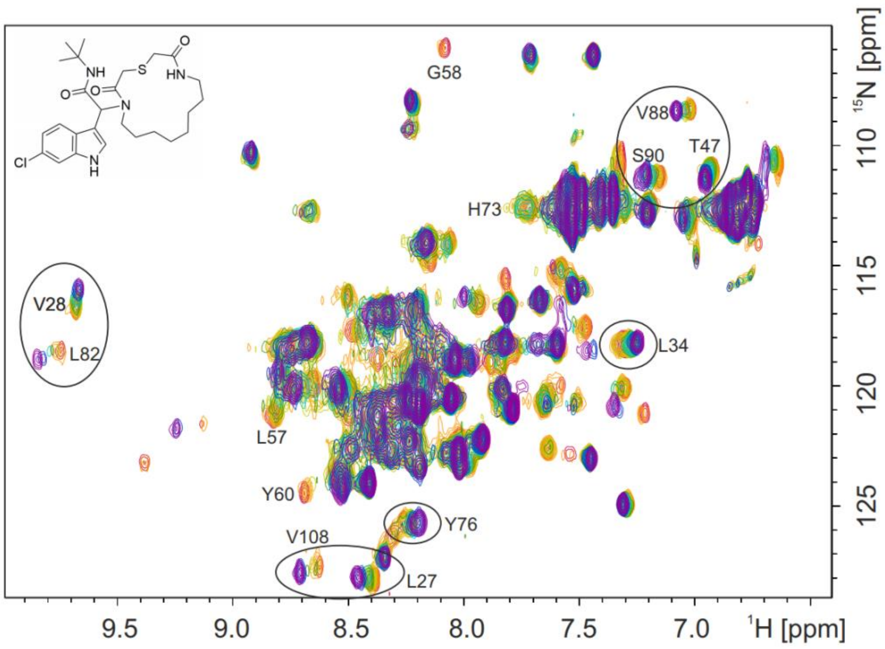

Figure S2. Overlay of ^{1}H-^{15}N-HSQC spectra of the reference MDM2 (red) and the titration steps with **2n** inhibitor. MDM2/**2n** ratios 4:1 (orange), 4:2 (yellow), 4:3 (green), 1:1 (light blue), 1:2 (blue), 1:5 (purple). Examples of most perturbed residues are labeled on the spectrum.

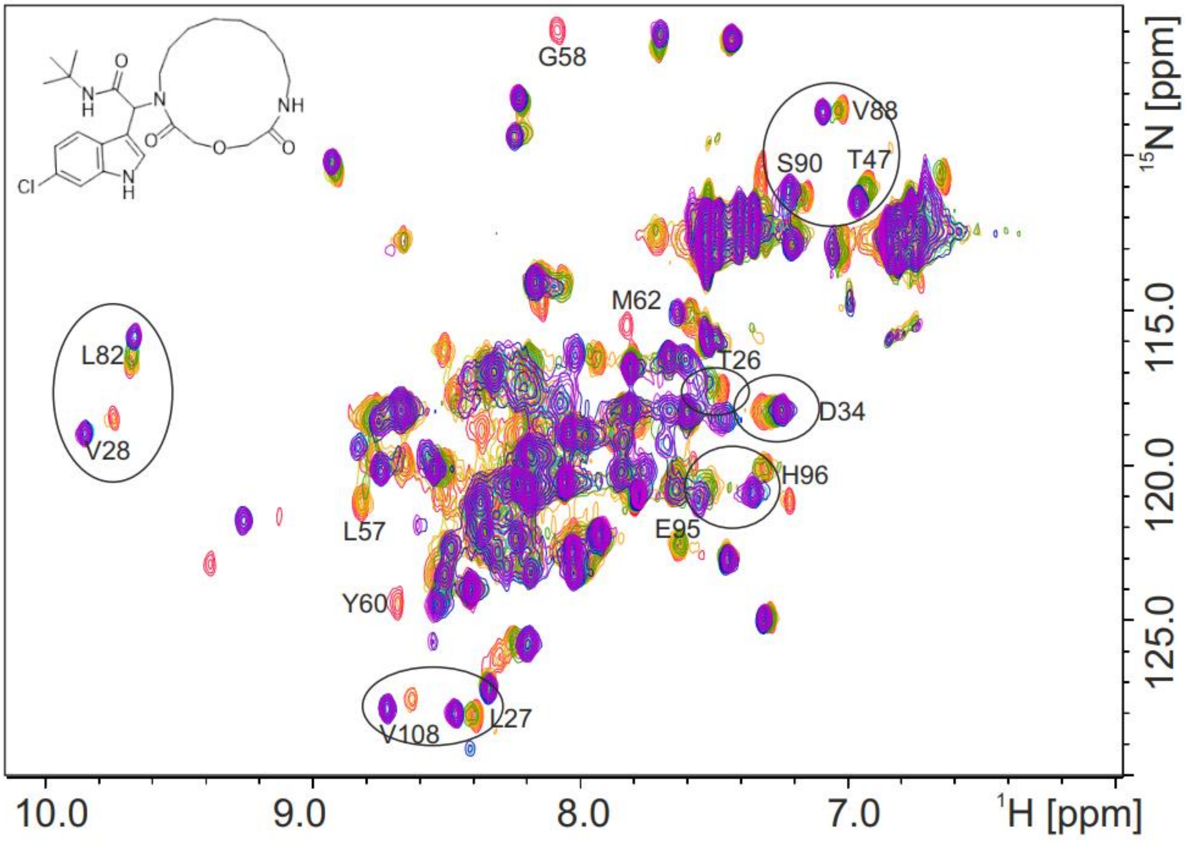

Figure S3. Overlay of ^{1}H-^{15}N-HSQC spectra of the reference MDM2 (red) and the titration steps with **2h** inhibitor. MDM2/**2h** ratios 6:1 (orange), 4:1 (yellow), 4:2 (green), 1:1 (light blue), 1:2 (blue), 1:5 (purple). Examples of most perturbed residues are label on the spectrum.[(5)]

Table S2. Final value of K_D was calculated as a weighted mean value of K_D's obtained for most perturbed residues which undergo fast chemical exchange.

Entry	Name	Structure	K_d MDM2 [μM]
1	**2g**	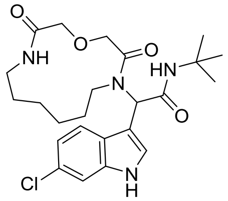	8.9±1.2
2	**2h**	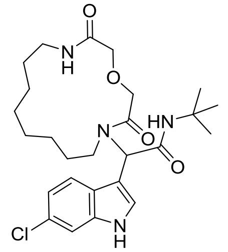	12.1±8.5
3	**2i**	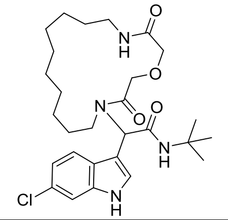	4.8±1.5
4	**2n**	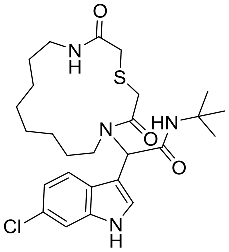	17.2±3.8

6. Computational modeling of macrocycles 2h, 2i and 2n

Computational modeling of compounds **2h**, **2i** and **2n** was performed using MOLOC software.[9] The crystal structure of the interaction of p53 peptide bound to MDM2 receptor (PDB ID 1YCR) was used for modeling.[10] The 2D structure of compounds **2h** **2i** and **2n** (*R*-enantiomer) was converted into a 3D structure and energy minimized in the absence of the receptor. Next, the 3D structures of the compounds were manually placed into the MDM2 receptor and the indole ring was aligned to the indole ring of W23 of the p53 peptide. The *tert*-butyl amide group was oriented into the F19 pocket of MDM2 as seen in other small molecule MDM2 cocrystal structures (PDB ID 3TU1, 3TJ2, 4MDN).[11] Afterwards, the macrocycles were energy optimized in the MDM2 receptor using the standard settings of the force field of MOLOC (Figure S4). The structures were rendered using PYMOL (the PyMOL molecular graphics system, version 1.2r3pre, Schrödinger, LLC).

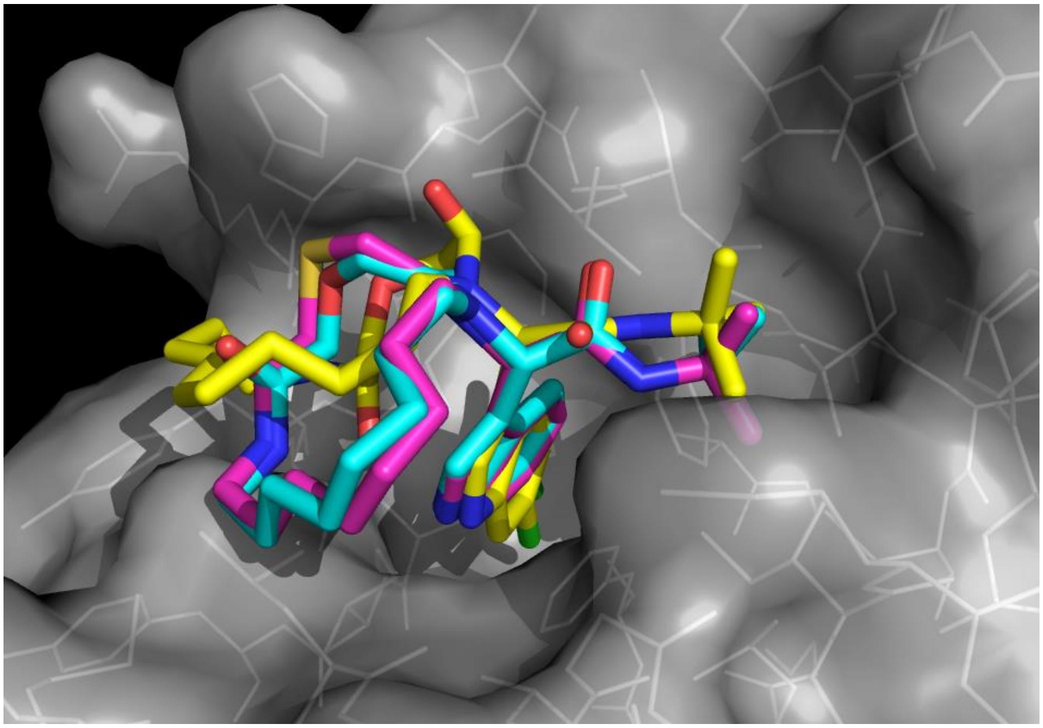

Figure S4. Modeling of the macrocycle **2h** (cyan sticks), **2i** (yellow sticks) and **2n** (magenta sticks) into the MDM2 receptor (PDB ID: 1YCR)

References

(1) Czarna, A.; Popowicz, G. M.; Pecak, A.; Wolf, S.; Dubin, G.; Holak, T. A. *Cell Cycle Georget. Tex* **2009**, *8*, 1176.

(2) Piotto, M.; Saudek, V.; Sklenár, V. *J. Biomol. NMR* **1992**, *2*, 661.

(3) Mori, S.; Abeygunawardana, C.; Johnson, M. O.; Vanzijl, P. C. M. *J. Magn. Reson. B* **1995**, *108*, 94.

(4) Stoll, R.; Renner, C.; Hansen, S.; Palme, S.; Klein, C.; Belling, A.; Zeslawski, W.; Kamionka, M.; Rehm, T.; Mühlhahn, P.; Schumacher, R.; Hesse, F.; Kaluza, B.; Voelter, W.; Engh, R. A.; Holak, T. A. *Biochemistry (Mosc.)* **2001**, *40*, 336.

(5) Madhavachary R.; Abdelraheem E.M.M.; Rossetti A.; Twarda-Clapa A.; Musielak B.; Kurpiewska K.; Kalinowska-Tłuścik J.; Holak T.A; Dömling A. *Angew. Chem. Int. Ed.* **2017**, *56*, 10725.

(6) Williamson, M. P. *Prog. Nucl. Magn. Reson. Spectrosc.* **2017**, *73,* 1-16.

(7) Fielding, L. *Prog. Nucl. Magn. Reson. Spectrosc.* **2007**, *51*, 219-242.

(8) Shuker, S. B., Hajduk, P. J., Meadows, R. P., and Fesik, S. W. *Science* **1996**, *274*, 1531–1534.

(9) Gerber P.R.; Muller K. *J. Comput. Aided Mol. Design* **1995**, *9*, 251.

(10) Kussie P. H.; Gorina S.; Marechal V.; Elenbaas B.; Moreau J.; Levine A. J.; Pavletich N. P. *Science* **1996**, *274*, 948.

(11) a) Huang Y. J.; Wolf S.; Koes D.; Popowicz G. M.; Camacho C. J.; Holak T. A.; Dömling A. *Chemmedchem* **2012**, *7*, 49. b) Bista M.; Wolf S.; Khoury K.; Kowalska K.; Huang Y. J.; Wrona E.; Arciniega M.; Popowicz G. M.; Holak T. A.; Dömling A. *Structure* **2013**, *21*, 2143.
